# Reintroducing face-to-face support alongside remote support to form a hybrid stop smoking service in England: a formative mixed methods evaluation

**DOI:** 10.1186/s12889-024-18235-0

**Published:** 2024-03-06

**Authors:** Nicholas Woodrow, Duncan Gillespie, Liz Kitchin, Mark O’Brien, Scott Chapman, Nai Rui Chng, Andrew Passey, Maria Raisa Jessica Aquino, Zoe Clarke, Elizabeth Goyder

**Affiliations:** 1 Sheffield Centre for Health and Related Research (SCHARR), University of Sheffield, Sheffield, UK; 2 Living Well Smokefree Service, North Yorkshire Council, York, UK; 3 MRC/CSO Social and Public Health Sciences Unit, School of Health and Wellbeing, University of Glasgow, Glasgow, UK; 4 School of Health, Leeds Beckett University, LS1 3HE Leeds, UK; 5 Population Health Sciences Institute, Faculty of Medical Sciences, Newcastle University, Newcastle upon Tyne, UK

**Keywords:** Smoking cessation, Hybrid, Remote, Equity, Service reorganisation

## Abstract

**Background:**

During the COVID-19 pandemic, United Kingdom (UK) stop smoking services had to shift to remote delivery models due to social distancing regulations, later reintroducing face-to-face provision. The “Living Well Smokefree” service in North Yorkshire County Council adopted a hybrid model offering face-to-face, remote, or a mix of both. This evaluation aimed to assess the hybrid approach’s strengths and weaknesses and explore potential improvements.

**Methods:**

Conducted from September 2022 to February 2023, the evaluation consisted of three components. First, qualitative interviews involved 11 staff and 16 service users, analysed thematically. Second, quantitative data from the QuitManager system that monitored the numbers and proportions of individuals selecting and successfully completing a 4-week quit via each service option. Third, face-to-face service expenses data was used to estimate the value for money of additional face-to-face provision. The qualitative findings were used to give context to the quantitative data via an “expansion” approach and complementary analysis.

**Results:**

Overall, a hybrid model was seen to provide convenience and flexible options for support. In the evaluation, 733 individuals accessed the service, with 91.3% selecting remote support, 6.1% face-to-face, and 2.6% mixed provision. Remote support was valued by service users and staff for promoting openness, privacy, and reducing stigma, and was noted as removing access barriers and improving service availability. However, the absence of carbon monoxide monitoring in remote support raised accountability concerns. The trade-off in “quantity vs. quality” of quits was debated, as remote support reached more users but produced fewer carbon monoxide-validated quits. Primarily offering remote support could lead to substantial workloads, as staff often extend their roles to include social/mental health support, which was sometimes emotionally challenging. Offering service users a choice of support options was considered more important than the “cost-per-quit”. Improved dissemination of information to support service users in understanding their options for support was suggested.

**Conclusions:**

The hybrid approach allows smoking cessation services to evaluate which groups benefit from remote, face-to-face, or mixed options and allocate resources accordingly. Providing choice, flexible provision, non-judgmental support, and clear information about available options could improve engagement and match support to individual needs, enhancing outcomes.

**Supplementary Information:**

The online version contains supplementary material available at 10.1186/s12889-024-18235-0.

## Introduction

Smoking rates in the United Kingdom (UK) have fallen rapidly since the publication of the Smoking Kills White Paper in 1998 [1], but smoking rates remain high in certain sectors of the population. The UK policy narratives on reducing smoking rates highlight the high smoking rates among people who work in routine and manual occupations and people who live in the 20% most deprived areas [2, 3]. Smoking rates are particularly high among people experiencing multiple disadvantage and social exclusion, e.g., people experiencing homelessness and people in minority communities [4]. The National Health Service (NHS) Core20Plus5 approach to reducing health inequalities [5] emphasises the need to focus on “priority population groups” such as, pregnant and new mothers, people with a long-term mental health condition (particularly people with anxiety, depression, and severe mental illness), people with drug and alcohol dependence, people with a learning disability, and people with long-term physical health conditions (particularly chronic respiratory disease, cancer, and cardiovascular diseases). Given the disproportionate rates of smoking for people experiencing multiple disadvantages [2–4] and given that smoking is a leading cause of preventable chronic illness [5], to reduce health inequalities, models for the provision of support to stop smoking [6] need to be better targeted to “priority population groups” [7, 8].

Community Stop Smoking Services (CSSS) are at the forefront of efforts to reduce smoking in the UK [9, 10]. They work by receiving referrals from primary and secondary care services, maternity services as well as self-referrals, and supporting service users to work towards stopping smoking. They provide regular support, including behavioural and pharmacological interventions, with the aim of supporting the service user to achieve a 4-week quit. It is well established that engagement with CSSS can lead to increased successful quit attempts [11–13]. However, the low uptake of and engagement with CSSS by priority population groups [14], suggests that CSSS could be made more accessible to meet the needs of such populations.

During the COVID-19 pandemic, restrictions on the provision of face-to-face support [15] resulted in CSSS moving to “remote” delivery models, i.e. online and phone-based provision [16–18]. There is evidence that remote approaches are an effective means of delivering stop smoking support [19–22]. Remote provision can increase the availability of stop smoking support, especially in rural communities [17]. It can also increase the accessibility of support by decreasing the time and costs required by service users to attend support sessions [16, 20, 23]. Some CSSS have reported that service users had positive perceptions of remote provision during the COVID-19 pandemic [9, 24]. However, concerns around remote-only provision in terms of its suitability to different population groups, if staff can provide support of the same efficacy over remote mediums, and if wide-scale remote provision is sufficiently resourced, have been noted [17].

This study reports on a Public Health Intervention Responsive Studies Team (PHIRST) formative evaluation (see Elwy et al. [25]) of the North Yorkshire Council Living Well Smokefree (LWSF) service. The LWSF service is commissioned by North Yorkshire County Council as part of their strategy to reduce smoking prevalence across the North Yorkshire region [26]. To continue operating during the COVID-19 pandemic, the LWSF service moved from face-to-face provision, which was the main method of engaging service users, to predominantly remote delivery, with support being typically delivered through voice calls (video calls were provided for those who requested this, but this had a much lower use) [18]. As COVID-19 restrictions eased, the LWSF service developed a “hybrid” approach that includes three service delivery modalities: (1) “Face-to-face”– support delivered in-person, (2) “Remote”– stop smoking support via voice or video calls; (3) “Mixed”– a flexible combination of both face-to-face and voice or video calls. The motivation for doing so was to retain the positive aspects of remote provision whilst ameliorating issues for service users who would have benefitted from face-to-face support. Further, a hybrid service was seen to offer an equitable option for the county’s population (which is relatively geographically dispersed), providing the potential to overcome differences in service users’ geographical proximity to clinics. North Yorkshire is one of the largest English councils by area, with a sizable rural population [27], and the LWSF service only has the equivalent of six and a half full-time staff members to provide support over this area. The local authority is among the least deprived in England, ranking 125th least deprived out of 152 upper-tier local authorities for the 2015 Index of Multiple Deprivation (IMD), although there are pockets of high levels of deprivation within the county [28].

Our study aimed to assess the benefits and challenges of reconfiguring the LWSF service to provide a hybrid mix of face-to-face, remote and mixed support options. We sought to explore the extent to which hybrid approaches are acceptable delivery models to service users and practitioners, are equitable and provide value for money. To do this we drew on elements from evaluation frameworks [29, 30] to help highlight key considerations from service users, service staff and service managers, and insights from service data. The evaluation design and primary questions that this project aimed to answer were co-developed with the LWSF service stakeholders as part of an initial Evaluability Assessment (see Additional file 1) [31]. The two evaluation questions were: (1) What are the strengths and weaknesses of the new hybrid approach to service delivery? (2) How could the hybrid service be adapted and improved?

## Methods

Our evaluation employed a mixed methods approach. We used interviews and focus groups to collect primary qualitative data which explored service staff and service user’s perspectives and experiences of the hybrid LWSF service, and existing quantitative data to explore service monitoring and outcome measures.

### Qualitative methods

#### Sample and recruitment

This research employed a purposive and convenience sample, which was designed to capture a range of perspectives, and which reflected the practical challenges of recruitment in real-world settings and in “live” health services [32, 33]. Eleven staff involved in the design and delivery of the LWSF service (including seven stop smoking advisors (SSAs), two specialist advisors, and two service managers) participated in four focus group interviews. Sixteen service users receiving support from the LWSF service were also interviewed. Our service user sample included 13 females and 3 males, and was predominantly white British (13 White British, 2 Mixed/Multiple ethnic groups, 1 Asian/Asian British). The majority of the participants received remote support (12 remote support, 2 mixed, 2 face-to-face). (See Addition file 2 for service user demographic/characteristic information). Recruitment and dissemination of study information was facilitated by the LWSF managers. Staff involved in the LWSF service were provided with information sheets and consent forms (see Additional file 2) and invited to participate. Service users were approached by their SSA and provided with a verbal description of the study, and a project information sheet if they expressed interest in participation. All participants were provided with time to read the information sheet and to ask questions about the study and their participation. All participants consented to their contact information being securely transferred to the research team. The research team contacted potential participants to further discuss the research and then followed up after a week to arrange interview times. All participants provided written informed consent for their involvement in the research which was electronically signed.

#### Data generation

All data collection took place via an online video call platform or through a telephone call. All focus groups and interviews were facilitated by NW. The focus groups ran between September 2022 and March 2023 and lasted between 60 and 70 min. The interviews were conducted between September 2022 and February 2023 and lasted between 25 and 35 min. We recruited fewer service user participants than originally intended due to recruitment challenges (*n* = 20). Similar perspectives around service experience were continually present in the participant’s accounts, but that is not to say a larger or more diverse sample would not have produced differing perspectives. Nevertheless, our sample appeared sufficient enough to provide valuable insights and data to answer our research questions and address our aims [34].

The focus groups and interviews followed semi-structured topic guides (Additional file 2) which explored perspectives and experiences around offering and receiving remote vs. face-to-face vs. mixed smoking support, facilitators and barriers of engagement through different provision modalities, and suggestions for future service provision and development. An effort was made to ensure all questions were asked. The topic guides, information and consent forms were reviewed for comprehension and additional avenues of exploration, and then revised through public involvement, engagement and participation work. This was supported by our LWSF service partners who recruited LWSF service users not involved as participants in the study to act as our public advisors.

Data management, confidentiality, right to withdraw and consent were verbally reaffirmed before data collection began. All focus groups/interviews were audio-recorded only, using an encrypted recorder, and transcribed verbatim by a university-approved transcriber. All transcripts were anonymised at the point of transcription, checked for accuracy by NW, and stored securely.

#### Data analysis

Qualitative data were analysed by NW and LK drawing on Braun and Clarke’s [35] thematic analysis approach. An initial coding frame was inductively developed based on initial readings of the transcripts; both NW and LK separately read a selection of transcripts, with initial thoughts and ideas being noted. Transcripts were re-read and coded using a line-by-line approach, where codes were generated inductively. The coding framework was revised following discussions between NW and LK, with the codes examined, merged, and grouped into potential themes and sub-themes. The revised framework (Additional file 2) was applied to the transcripts by NW, and LK separately coded a selection of transcripts to check for accuracy and consistency. NW and LK discussed the coding and reviewed and refined the themes following this. Through the analysis five key themes were generated: (1) Reach of the service to priority service user groups, (2) Perceived effectiveness of the stop smoking intervention, (3) Accommodation of service users’ needs, (4) Implementation of the hybrid service, and (5) Maintenance of the hybrid service. Transcripts were coded using the qualitative data management software system NVivo 12.

### Quantitative methods

#### Service use and effectiveness

The LWSF service routinely monitors service usage and quitting outcomes in terms of 4-week quits [36]. Data is recorded on QuitManager (North 51, Nottingham, UK), an online database system for recording information on service users and intervention characteristics. The LWSF team regularly inspect these data by generating summary statistics of service use and outcomes, stratified by priority service user groups, geographic areas, and source of referral. With the introduction of the new hybrid service, this monitoring was extended to include variation by mode of contact with the service, i.e., differences in usage and outcomes depending on whether a service user initially chose the remote, face-to-face or mixed service pathways. For reasons of practicality, the recording of data on the pathway chosen by the service user was based on their preference expressed at first contact with the service. If a service user initially chose the remote or face-to-face options and then it was agreed that they would receive a mixture of contact modalities, their recorded pathway was changed to the mixed option. However, if a service user chose the face-to-face option and happened to need a remote session because they were unable to attend face-to-face, then they were still recorded under the face-to-face option. The service began to collect quantitative data in this structure to enable monitoring of differential use of the three pathways in September 2022. A summary of the 6 months of data provided by the service is available in Additional file 3.

Due to the early stage of hybrid service implementation and the limited data available, statistical analysis of the difference in quit rates was not possible due to the small sample sizes for the different modalities. Therefore, only descriptive statistics are presented (see Tables 1 and 2).

**Table 1:** Descriptive statistics of service usage in the period September 2022 to February 2023

	Total	Phone support	Face-to-face support	Mixed support
Referred into service	892			
Accessed the service	733	669 (91.3%)	45 (6.1%)	19 (2.6%)
Set a quit date	398	367 (92.2%)	23 (5.8%)	8 (2.0%)
Achieved a 4-week quit	303	279 (92.1%)	17 (5.6%)	7 (2.3%)
Number and Percentage of people				
Who accessed the service and who achieved a 4-week quit	303 (41.3%) out of 733	279 (41.7%) out of 669	17 (37.8%) out of 45	7 (36.8%) out of 19
Who set a quit date and who achieved a 4-week quit	303 (76.1%) out of 398	297 (76.0%) out of 367	17 (73.9%) out of 23	7 (87.5%) out of 8

#### Value for money of face-to-face services

The evaluability assessment process identified a need to estimate how the new hybrid service affected the value for money of the LWSF service from the provider’s perspective. Value for money is normally assessed using a metric of the “cost per 4-week quit” generated by the service. The evaluation aimed to estimate the value of reintroducing face-to-face delivery by estimating the additional monthly cost to the service of providing face-to-face appointments (that could be used by service users in the face-to-face or mixed pathways) and dividing this cost by the number of 4-week quits achieved by service users in the face-to-face or mixed pathways. The definition of cost components was discussed with the service, resulting in the costs being based on the cost of venues in which to hold face-to-face clinics, and the cost of travel and parking for service staff. The costs of carbon monoxide (CO) monitoring were not included due to the low rate of CO monitoring at the time of the evaluation and the relatively modest cost of the consumables involved in CO monitoring. The cost data used in the evaluation is available in Additional file 3.

### Mixed methods synthesis of findings

Drawing on an “expansion” approach [37] and complementary analysis [38], findings from the qualitative themes and quantitative elements were synthesised to give a five-part structured answer to the two evaluation questions. We present our findings using the 5 key themes generated from the qualitative analysis as headings: (1) Reach of the service to priority service user groups, (2) Perceived effectiveness of the stop smoking intervention, (3) Accommodation of service users’ needs, (4) Implementation of the hybrid service, and (5) Maintenance of the hybrid service. Where appropriate, we have used available quantitative data to complement the qualitative findings. Our synthesis integrated findings across the interviews with service users and service staff, the discussions with the service management team as part of the formative evaluation process, and the quantitative data provided by the service to inform the evaluation. The qualitative data provided context to the quantitative findings, which is important because the quantitative findings were a snapshot of the new hybrid service at an early stage in its implementation.

Research question 1 (What are the strengths and weaknesses of the new hybrid approach to service delivery?) is addressed across the first four themes. Research question 2 (How could the hybrid service be adapted and improved?) is predominantly addressed in theme five, but other findings are presented where relevant across the first four themes.

### Ethics

Ethical approval for the study was granted by the Sheffield Centre for Health and Related Research (SCHARR) ethics committee at the University of Sheffield, application reference number: 049146.

## Results

Overall, there was a general preference for remote over face-to-face provision by service users and staff, with over 90% of service users who accessed the service selecting remote support (Table 1). For those who set a quit date, phone support was found to have similar, but slightly higher, 4-week quit outcomes compared to face-to-face support– 76% (279 quits out of 367 quit dates set) vs. 73.9% (17 quits out of 23 quit dates set), but lower than mixed support– 87.5% (7 quits out of 8 quit dates set). However, the early stage of hybrid service implementation, and the small sample sizes on which these comparisons are based, mean that there is too much uncertainty to be able to draw conclusions about differences in quitting outcomes between the different modalities (Table 1).

### Reach of the service to priority service user groups

Remote provision was described by service staff as enabling a much wider service offering and facilitating greater access and support for different populations, including for people who were often “geographically excluded” in rural areas far from available clinics, those who struggled to access face-to-face appointments due to mental or physical health issues, and those unable to attend due to time requirements and employment commitments:

*“I’m speaking to people I never would have spoken or had a face-to-face with because it wouldn’t have been possible for me to go there. So it has made the service more accessible to those hard to reach rural groups and people who couldn’t attend because of their employment situation and so on.” (FG1– service staff)*.

There was a suggestion from service staff that more flexible ways were needed to engage with the current smoking population, and that a hybrid service can help to facilitate this:

*“We’re at the harder to reach smokers, we’re at the stage of the difficult smokers now, so if anything we need to be even more accommodating to them, this [hybrid service] can solve that really.” (FG3– service staff).*

*The modality selection preferences for all service users who set a quit date was 92.2% phone support, 5.8% face-to-face support and 2.0% mixed support (see table *1). *This overall pattern was reflected in the priority population groups:* pregnant service users (98.2%, vs. 0% face-to-face and 1.8% mixed support), people with mental health conditions (93.4%, vs. 5.1% face-to-face and 1.5% mixed support) and people with long-term physical health conditions (91.2%, vs. 5% face-to-face and 3.8% mixed support) (Table 2).

*“It has helped not actually having to have to visit somebody face-to-face…it’s a lot more beneficial to people who have anxiety and mental health issues like me.” (IN12– service user)*.

**Table 2:** Quit dates set by priority groups in the period September 2022 to February 2023

	Set a quit date						4-week quit outcomes			
	Number of people	Percentage of people	Phone support	Face-to-face support	Mixed support		Number of 4-week quits	Phone support	Face-to-face support	Mixed support
Pregnant service users	55	14%	54 (98.2%)	0	1 (1.8%)		42 (76.4%)	41 (75.9%)	0	1 (100%)
People with mental health conditions	136	34%	127 (93.4%)	7 (5.1%)	2 (1.5%)		99 (72.8%)	92 (72.4%)	6 (85.7%)	1 (50.0%)
People with long-term physical health conditions	159	40%	145 (91.2%)	8 (5.0%)	6 (3.8%)		119 (74.8%)	109 (75.2%)	6 (75.0%)	4 (66.7%)

### Perceived effectiveness of the stop smoking intervention

#### Privacy and openness

For many service users, phone support was spoken of as enabling more openness in discussions, typically through perceived partial anonymity and a greater level of privacy due to participation from known places of comfort. Further, the perceived protections that remote provision offers acted to reduce potential stigmas (e.g., around smoking during pregnancy) and anxieties (e.g., having to attend clinics in person for people with mental health issues) associated with attending face-to-face clinics, and was noted as important in facilitating engagement and openness during appointments:

*“It [remote provision] means people don’t see you going in when you can do it over the phone, so it’s better when you’re pregnant so you know you won’t be judged by anyone.” (IN15– service user)*.

### Carbon monoxide (CO) monitoring

A challenge of remote provision, that some SSA saw as linked to service user engagement and motivation, was the inability to conduct CO monitoring to validate quits. This was commented on as reducing service user accountability, and removing a tangible measure of success, as CO monitoring was seen as something that could motivate service users:

*“It’s almost like when people go to slimming world or weight watchers, that scale gives them accountability. And they think that the CO reader is that accountability, it’s like ‘ooh let’s see what I can blow this week’. And it does help motivate people quite a lot” (FG1– service staff)*.

However, there were mixed perspectives from service users around the use of CO monitoring, with some unaware of it being offered, some using and seeing it as a motivating and engaging feature of support, whilst others were indifferent about its use.

### Barriers and facilitators to rapport development

There were mixed views over the ability to develop rapport and therapeutic relationships via remote means. Some SSA and service users suggested it was easier and quicker to develop a rapport face-to-face, due to the interpersonal connection such sessions provided. Despite this, many participants noted they were able to develop effective relationships and engagement with service users/SSA through remote means only. A key benefit of mixed provision was the ability to have initial face-to-face contact with a SSA which was noted as facilitating the development of rapport for some:

*“I liked it the way it happened with me, where I see them at the start, and then just phone after…then you already know who it is, so, and with phone calls then, it’s still quite personal, because it’s not just a voice, know what I mean.” (IN16– service user)*.

Interestingly, there was a suggestion from some SSA that, for some service users, remote provision can result in poor engagement with sessions, due to remote support not permitting focus and attention:

*“The difficulty we’ve had with telephone support for a lot of the time was that you’d ring and they were like out doing their shopping. I mean in COVID it was different because a lot of people were stuck at home…but sometimes now, you aren’t getting their full attention…They’re not really like engaging with you.” (FG2– service staff)*.

### Accommodation of service users’ needs

Having a hybrid approach was consistently noted as providing the flexibility and accommodation to meet the eclectic needs of service users, to enable support to fit into people’s personal and work lives, and to remove “barriers” around service engagement:

*“It’s like, booking time off work for an appointment and stuff like that, whereas doing it remotely, I could just do it at work, just saying I’ll be back in five minutes I’ve got an appointment on the phone…It’s the time and having to go to appointments, know what I mean, it’s just easier on the phone.” (IN3– service user)*.

Whilst the majority of service users were selecting remote over face-to-face or mixed provision (Table 1), there was still a perceived need and want for face-to-face options from some service users. Further, offering mixed support (typically experienced as being able to move from face-to-face to phone support), was particularly valued at permitting the continuation of support in response to the complexities of everyday life:

*“It’s the best of both worlds really, so it’s made it all more accessible for me…, because when I couldn’t make a meeting due to childcare issues, I could still get my support that week over the phone.” (IN4– service user)*.

### Supportive and flexible approaches

It was consistently clear that each service user was different, and thus required support and approaches to meet individual needs and preferences. Having the ability to choose the type of provision received, and having flexibility in support, was valued as an important aspect of engagement which contributed to perceived service satisfaction:

*“Having that flexibility in getting support means I can get that support when I need it, it’s like allowing me to make the most of my determination before it’s gone.” (IN6– service user)*.

The perception of non-judgmental supportive relationships from service staff was noted as crucial in engagement and rapport building for service users. It appeared that the skills of staff to develop relationships, and the levels of care, compassion, and personalised support they provided, helped engagement irrespective of the modality of support:

“*I never felt judged, and that’s what I was scared of [being pregnant], but straight away even on the phone [name] made me feel at ease, just really friendly. That helped me open up, because we just got on*”. (IN15– service user)

### Implementation of the hybrid service

#### “Quantity vs. quality” of quits

Generally, the SSAs and service managers noted that remote provision was a much more “efficient” use of time, compared to purely face-to-face provision, in terms of increasing SSA caseload capacity and the number of service user contacts they could have. However, due to the nature of remote provision, quits were self-reported and not validated by CO monitoring. In light of the reported lack of national or local requirement for services to achieve CO validated quits, the LWSF service were prioritising offering service users’ choice and flexibility in the support they received. Any national or local change in requirements around achieving CO validated quits was noted as requiring a change in how the hybrid service would be delivered, and the proportion of remote and face-to-face provision offered:

*“If you wanted to get to as close to 100% CO validation as possible, you’d need to put a lot of face-to-face clinics on, and then you’d need to not offer as much remote, and that would cut down on your numbers… it’s a difficult balance to strike between getting people in face-to-face, getting CO validation rates up, but seeing the amounts of people we need to, and having flexibility to see people.” (FG4– service managers)*.

Therefore, achieving CO validated (“quality”) quits was seen to be balanced against the number (“quantity”) of quits and service user engagement that could be achieved through remote provision.

#### Managing increased caseloads

Offering primarily remote provision was described by some SSA as resulting in, at times, considerable workloads and extremely large caseloads, specifically during “peak” periods. Further, many SSA felt their roles went beyond stop smoking support to various aspects of social and mental health support, which was described as “draining”:

*“A lot of people talk about their problems during their phone calls, and actually, that can be very draining as well.” (FG2– service staff)*

These issues were particularly pertinent during the COVID pandemic and were noted to have been exacerbated by the consecutive “back-to-back” nature of phone support sessions, and the lack of formal and informal team support accessible through complete working from home:

*“[It’s] a little bit isolating. Because you’re working from home, you don’t have any colleagues that you see every day, you’re not having those corridor talks or those five minutes for a coffee in the canteen… being able to sit and have a chat with somebody, and a moment to sorta say ‘oh did you find that!’ and ‘oh I’ve been struggling with that!’. And a lot of that is missing.” (FG1– service staff)*

### Maintenance of the hybrid service

#### Data-led service delivery decisions

To inform future service adaptations to the needs of the local population, it was noted that data on the hybrid service was needed over a longer period of implementation. This would better determine the relative effectiveness of the remote, face-to-face and mixed pathways (in terms of the number of people setting and achieving 4-week quits) and how this varied by population demographics (e.g., ages, genders, ethnicities, geographies) and priority groups (e.g., maternity, mental health, substance misuse). See Table 2, and Additional file 3 for emerging data around variations in quit outcomes between the different pathways for different priority groups.

We estimated that the additional cost of providing a face-to-face option in the period September 2022 to February 2023 was £692 per month. In the average month from September 2022 to February 2023, 149 people were referred to the LWSF service. Using the percentages from Table 1, the service could expect 4 of these people to achieve a 4-week quit using either the face-to-face or mixed pathways. Dividing the total cost by the total quits gave a cost per 4-week quit from the additional face-to-face option of £175. (See Additional file 3 for further detail and breakdown around the calculation of return on investment for the face-to-face offering). Whilst information on the “cost-per-quit” for each of the three pathways was noted as important, offering service users a choice of pathways was perceived as crucial and potentially more important than any differences in the value for money among the pathways provided.

There were discussions around offering all service users a choice around pathway options and providing a more tailored and targeted approach for different populations and priority groups. This again was discussed in terms of a “quantity vs. quality” perspective, with a perceived need for targeted support for some smokers, but reflection that this could be a more time and resource-intensive approach. National and local priorities around guidance on what the LWSF service should be offering, as well as ongoing data collection, were suggested to be the key influences on the promotion and adaptation of support pathways going forward.

#### Improving awareness of the hybrid service

The most consistent suggestion for service improvement was for better service information and knowledge to be available, advertised and disseminated, specifically around the hybrid offering and the flexibility of provision:

*“I think most people think that it is just like face-to-face appointments and if people are working they struggle to get to appointments or if they’ve got young kids. So, if it’s made clear that there are both face-to-face appointments and over the phone whichever works best for them, that could make a big difference.” (IN1– service user)*

Several service users who were “referred in” from primary and secondary services noted receiving little information about the LWSF service, in terms of the modalities of support available, how support was provided and the flexibility of support. This information was noted as important in reducing anxieties and encouraging engagement.

## Discussion

The LWSF service’s shift towards remote provision was seen to have enabled a greater level of service accessibility, removing barriers to support for those who were previously excluded, unable or struggling to attend and participate in face-to-face provision. The re-introduction of face-to-face provision, and offering of mixed support, to form a hybrid approach was seen to expand service user choice and access. For some staff, remote provision was noted to have produced increased efficiency in terms of the number of people receiving support, but also increased caseloads. There was a perceived trade-off between the “quantity vs. quality” of quits, where face-to-face contact was seen to produce a smaller number of “higher quality” CO validated quits vs. the greater quantity of quits that could be achieved through more “efficient” remote provision. Tailoring support pathways to specific populations was noted as potentially more resource-intensive than offering a flexible choice of support options to all, but it was also noted that this might be required in order for the service to effectively support these populations.

Personal contact, perceived connection and interpersonal relations between service users and providers have consistently been noted as important in engagement and treatment adherence [15, 16, 39] and in supporting, encouraging and motivating health-promoting behaviours [20, 40]. Remote provision was noted as beneficial in engaging priority groups by removing barriers around engagement, such as stigma, and fear of judgement [41], which can facilitate openness during sessions [42]. It was suggested in the accounts of the SSA that remote provision, especially now post-COVID lockdown policies, when people were not restricted to their homes, was resulting in lower levels of engagement for some service users. It has been noted in previous work that remote sessions are less engaging, and that service users spend more time participating in face-to-face provision than online/remote sessions [23]. Thus, remote sessions may have greater accessibility, but at the potential cost of less engagement for some. In our study, whilst there was a suggestion that developing effective relationships through remote provision could be more difficult [20, 43, 44], it was still achievable. Regardless of the modality of care, our service user participants discussed the importance of a supportive therapeutic relationship upon their engagement, with non-judgmental and personalised approaches from service staff facilitating rapport building.

There does not appear to be a single approach that will universally suit all service users, but a key theme evident in our study was how valued a flexible, patient-centred approach was. This echoes findings from the wider literature where there is a consistent recommendation of a patient-centred approach (for example, the recent calls for personalisation in health care [45]), and where the modality of care is designed, chosen and delivered in conjunction with service users [16, 44]. It is important to note that despite flexibility and choice being valued by service users, some populations may require more targeted or tailored ways of provision (e.g., [46, 47]). Such considerations around offering “flexible choice” or “targeted” provision are crucial for services in the establishment, adaptation and promotion of support. Our results suggest that whilst there were similar 4-week quit outcomes for priority groups (see Table 2), there were some variations in quit outcomes between the different pathways for different groups (e.g. a higher percentage of 4-week quits were achieved with face-to-face support (85.7%, *n* = 6) compared to phone support (72.4%, *n* = 92) for people with mental health conditions (see Table 2). However, the short time that the LWSF hybrid service had been in operation and the small sample sizes in some subgroups leads to high uncertainty in conclusions. Longer-term data around the outcomes of different pathways for different populations, will inform and guide services around designing, targeting and tailoring provision to best support and engage service user engagement and access.

Misconceptions of what smoking services offer, their proximity and how they can be accessed have been noted as barriers to engagement [48]. Such issues were commented on in our study, with service user participants highlighting a general lack of awareness around available service provision options before and during their referral. Therefore, ensuring that accurate knowledge of CSSS and their offer is received by potential service users is important, and may facilitate initial engagement.

Similarly, despite mixed awareness and perceptions from service users of CO testing, promoting this may be beneficial in engagement and encouraging uptake of face-to-face appointments by providing an additional motivation via a tangible marker of success [49]. Additionally, there is the ability for CSSS to employ remote CO monitoring (e.g., via the home delivery of devices and undertaking self-reported or video-monitored checks, undertaking home visits to complete tests, or organising verification at local clinics/chemists [50–53]. However, conducting remote CO verification of smoking has associated challenges, including issues around digital inequalities for service users, the accuracy of devices and recording [50], and crucially the costs of remote CO devices for CSSS [54]. Nevertheless, if remote support is to continue to be offered by CSSS and primarily selected by service users, it may be important for CSSS to consider strategies around delivering remote CO monitoring.

### Strengths and limitations

The main strength of our study is the formative evaluation approach that was designed to identify potential and actual influences on the progress and effectiveness of implementation efforts from the point of view of informing future service adaptations and responses [55]. Our formative evaluation was preceded by an Evaluability Assessment process in which the aims, objectives, and design of the subsequent evaluation were co-developed through a participatory process with service stakeholders (see Additional file 1) [56]. Evaluability Assessment is a rapid, systematic, and collaborative way of deciding how a programme or policy can be evaluated [31]. Our formative evaluation approach enabled us to deliver emerging findings back to the LWSF team, allowing data to help inform, improve and adapt the service during the evaluation process [57]– for example, around the value of more clearly presenting the hybrid offering and flexible options for potential service users.

The main limitation is that our study was based on only one stop smoking service in England, which was at an early stage of implementing its new hybrid service offering. The evaluation occurred under “real life” conditions, and thus there was no randomisation of service users to groups of remote, face-to-face or mixed provision, with service users selecting which provision mode they preferred at the start of their treatment. We worked with our LWSF project partners to try and represent the heterogeneity of participants the service engages with, but due to the nature and challenges of our recruitment methods, we were only able to sample service users opportunistically. Thus, our findings reflect the perspectives and experiences of the service users who had engaged and nearly completed their treatment. Future work could explore the experiences and perspectives of those who disengaged from support, or employ a targeted outreach approach to explore the experiences of marginalised populations often excluded from CSSS (e.g. the most disadvantaged groups [58, 59], such as homeless populations [60]).

## Conclusion

The findings of our study add to the evidence showing high levels of acceptability around remote [15, 61, 62] and flexible mixed provision [23], and shows the potential of hybrid treatment options for people trying to stop smoking [16, 23]. Our study illustrates how the establishment of hybrid support can provide an opportunity for services to assess which types of users in their local populations might benefit from remote, face-to-face or mixed options, as well as to direct their approaches and resources accordingly. Ensuring that CSSS provide choice around their support, flexible delivery of provision, non-judgmental support, and that there is clear understanding around what provision they provide, may contribute to engagement and successful outcomes through enabling preferred and beneficial support when needed.

### Electronic supplementary material

Below is the link to the electronic supplementary material.

Supplementary Material 1

Supplementary Material 2

Supplementary Material 3

## Data Availability

The qualitative datasets generated and/or analysed during this study are available from the corresponding author on reasonable request, and subject to approval from the Sheffield Centre for Health and Related Research (SCHARR) ethics committee at The University of Sheffield. The quantitative data provided by the LWSF service to inform the evaluation is summarised in the results section and in the supplementary material– Additional file 3. The data on which these summaries are based is not possible to make available due to the risk of disclosing individual service user identities. For the purpose of open access, the author has applied a Creative Commons Attribution (CC BY) licence to any Author Accepted Manuscript version arising.

## Abbreviations

CO: Carbon monoxide
CSSS: Community Stop Smoking Services
FG: Focus group
IN: Interview
LWSF: Living Well Smokefree
NHS: National Health Service
PHIRST: Public Health Intervention Responsive Studies Teams
SSA: Stop smoking advisor
UK: United Kingdom
