# Supplementary material for: Reintroducing face-to-face support alongside remote support to form a hybrid stop smoking service in England: a formative mixed methods evaluation
Source: BMC Public Health. 2024 Mar 6;24:718. doi: 10.1186/s12889-024-18235-0 (PMC10916048; doi:10.1186/s12889-024-18235-0)
Supplement: Supplementary file 1 — Supplementary Material 1 [file 12889_2024_18235_MOESM1_ESM.pdf]

North Yorkshire County Council ‘Living Well Smokefree’: Evaluability Assessment Report

PHIRST Fusion

September 2022

Summary

'Living Well Smokefree' (LWSF) is North Yorkshire County Council's programme to tackling smoking in the region. It has been running since 2021 and during the Covid-19 pandemic, it adapted into a hybrid model offering both remote (virtual) and face-to-face services. In 2020, NOLB Fife approached PHIRST for evaluation support. An evaluability assessment (EA) was facilitated by PHIRST Fusion for NOLB Fife from December 2021 to May 2021. A theory of change (ToC) for LWSF was co-developed with LWSF stakeholders and a feasibility evaluation study was agreed as the aim of the PHIRST Fusion evaluation. This report documents the EA process, presents the LWSF ToC and describes a list of evaluation design options and recommendations for the LWSF evaluation which focuses on learning about the delivery of services and recipients' experiences. Options recommended include relying on existing NOLB monitoring & evaluation (Option 0); Summative Process evaluation (Option 1); Formative Process evaluation with an interim check-in point (Option 2); and Formative Process evaluation with quarterly check-in points (Option 3). Option 2 is recommended to strike a pragmatic balance between programmatic learning and evaluation resources available.

Table of Contents

Summary 2

Table of Contents 3

1 Introduction 5

Background to the PHIRST scheme & LWSF study 5

Programme context 5

Impact of Covid-19 5

2 Rapid Review 6

3 Evaluability Assessment process 9

Workshop 1 9

Workshop 2 9

Workshop 3 10

Theory of Change 10

4 Evaluation design considerations 10

Aim & evaluation questions 11

Data 11

5 Evaluation options 12

Option 0: Existing M&E 14

Option 1: Summative Process Evaluation 14

Option 2: Formative Process Evaluation (Mid) 19

Option 3: Formative Process Evaluation (Quarterly) 19

6 Recommendation 20

Time period of data to be used for the evaluation 20

Development and qualitative description of the theory of change 20

Statistical descriptions of service outputs and outcomes 20

Reflective meeting on evaluation findings together with the service 20

Modelling and the calculation of cost per quit 21

Public involvement and engagement 21

Involving past clients in advising on how to best collect data from current clients 21

Involvement and engagement of LWSF staff in the evaluation 21

Service implementation and data recording 21

Readiness to reflect on and adapt service implementation 21

Supporting the qualitative investigation 21

Supporting the quantitative investigation – data processing and flows to ScHARR 22

References 23

Table 1 Summary of workshop dates & agenda 9
Table 2 Overview of LWSF evaluation options 13
Table 3 Important quantitative data fields 15
Table 4 Qualitative data to be collected 18

1 Introduction

Background to the PHIRST scheme & LWSF study

This evaluation initiative is funded by the National Institute for Health and Care Research (NIHR) and undertaken by the Public Health Intervention Responsive Studies Team (PHIRST) Fusion team. The PHIRST Fusion team's approach to evaluation follows a 5-step process: brokerage, work allocation, research, reporting & knowledge mobilisation, and continuous improvement, which includes evaluability assessment methodology and embedded research with local government practitioners. The Living Well Smoke Free Project (LWSF) was allocated to PHIRST Fusion in August 2021. This report describes an Evaluability Assessment of LWSF conducted between December 2021 and May 2022.

LWSF is an intervention offered in North Yorkshire since October 2021. It is a hybrid service programme comprising online and face-to-face elements. LWSF aims to offer a more flexible service for service users resulting in higher quit numbers/rates and improved rural access; ability to resume face-to-face interventions and CO monitoring with a focus on priority groups and key points of quit journeys; a more cost-effective service model; and a flexible way of working.

Programme context

Community stop smoking services are at the forefront of efforts to reduce premature death and disability due to smoking in the UK (ASH, 2022). 'Living Well Smokefree' (LWSF) is North Yorkshire County Council's programme to tackle smoking in the region. There are approximately 70,000 smokers within North Yorkshire, with higher rates being found in areas of greater deprivation, and worryingly, above national average rates of smoking in priority risk groups (such as during pregnancy and in populations with mental health conditions) (North Yorkshire tobacco control strategy, 2015). Working with people over the age of 12, the LWSF service offers one-to-one support over a 6-12 week period (one session per week) in an effort to achieve a Russell Standard quit (West, 2005). Support is provided to help service users set a quit date, and to manage cravings/withdrawal symptoms. Support includes pharmacotherapy (nicotine replacement therapy (NRT) medication vouchers provided by prescription), CO (carbon monoxide) monitoring, and every client has a named key worker (smoking cessation adviser) who employs Motivational Interviewing principles and techniques to increase self-efficacy to reduce smoking.

Impact of Covid-19

Due to the COVID-19 pandemic, to continue to support individuals wishing to quit smoking, the North Yorkshire Living Well Smokefree (LWSF) service moved to remote delivery with support being delivered in virtual formats. LWSF have seen remote delivery as having benefits (increased accessibility for rural communities), as well as challenges (difficulty in achieving/recording CO validated quits). Therefore, as COVID-19 restrictions have eased LWSF has developed a hybrid approach, combining elements of face-to-face and virtual delivery to better target different priority groups. This hybrid approach included three service delivery methods (face-to-face only, online-only, and a blend of face-to-face and online).

This study aims to explore the implementation of this hybrid smoking cessation model, and to assess the effectiveness and acceptability of the different delivery methods, in order to inform future service delivery

2 Rapid Review

We undertook a rapid literature scope to explore feasibility studies of smoking cessation services (including, blended/hybrid/flexible/tailored services, and online/phone/face-to-face approaches). Data was initially searched through google scholar. Initial search terms around 'smoking cessation services', 'feasibility' and 'delivery models' (e.g., blended/hybrid/flexible/tailored) were used. Relevant data was extracted from the returned results:

- 'Blended' = usually a specific intervention when some specific content is delivered face to face and other specific content is delivered online
- 'Hybrid' = the same service can be delivered in different formats/locations
- 'Flexible' = where the client has a choice of intervention delivery mode
- 'Tailored' = any service designed for and delivered to a specific patient group

<u>Service user perspectives</u>

Generally, work exploring service users' perceptions of remote smoking cessation provision found positive perspectives, experiences and high levels of acceptability, as well as preferences for engagement in remote provision modalities in the future (Mahoney et al., 2021). There were comparable and high levels of acceptance for both voice and video call approaches from service users (Kim et al., 2016; Richter et al., 2015). Kim et al. (2018) reported a slightly greater engagement in treatment (and higher maintenance of smoking abstinence) from video-call interventions over voice-call interventions. They also found similar, and high, levels of acceptability from participants between voice and video call approaches, but issues in the feasibility of video calls were noted (e.g., older and more deprived populations did not have technological access or knowledge for this approach). For some groups, remote provision may increase access to support, and thus be a feasible option (e.g., engaging rural communities (Byaruhanga et al., 2020; Carlson et al., 2012)), helping overcome issues such as travel time and costs for face-to-face support (Tall et al., 2015; Byaruhanga et al., 2020). Indeed, remote provision was perceived as acceptable by rural communities (Carlson et al., 2012), with video call models being a feasible option for smoking cessation services for rural populations (Byaruhanga et al., 2020).

However, remote provision may not be feasible for all types of support and may not be acceptable to all service user groups (Appleton et al., 2021). Remote provision may be less suited to/effective with populations with more complex health conditions and exacerbating health issues (e.g., pregnant women) and comorbidities (substance use and mental health issues) (Siemer et al., 2020; Appleton et al., 2021). Indeed, Ashton et al. (2015) reported much lower cessation rates for people with mental health issues who received phone

support only vs face-to-face and phone support. However, if provision is appropriate and designed for specific populations, remote provision can be acceptable, feasible and useful (e.g., Joyce et al., 2021; Bennet et al., 2016; Ashton et al., 2015). For example, Joyce et al. (2021) reported high levels of feasibility and acceptability of several components of a 'technology-supported, incentive-based smoking cessation program' for pregnant women, including the feasibility and strong acceptability of video chat observed cotinine testing. The importance of flexible approaches to engage different populations was evident (e.g., those in deprived areas (Ormston et al., 2015), pregnant women (Bennett et al., 2014; Joyce et al., 2021), people with severe mental illness (Ashton et al., 2015)). More important than a specific population, maybe the need for, where possible, a flexible and patient-centred approach, where the modality of care is designed, chosen and delivered in conjunction with service users (Vera San Juan et al., 2020; Ritchie et al., 2007). Having a mix of approaches to draw on may be beneficial in engaging and providing support for priority groups (Mahoney et al., 2021; Appleton et al., 2021). Blended approaches have been noted to have a generally positive experience (Siemer et al. 2020).

- Benefits of remote provision for participants included convenience (time/cost/effort), comfort and safety (Mahoney et al., 2021; Vera San Juan et al., 2021; Vinci et al., 2022). Video-call over voice-calls were noted as being more personal (Mahoney et al., 2021). Remote provision was seen as potentially beneficial for more 'functional' appointments (e.g., check-ins (Vera San Juan et al., 2020; Appleton et al., 2021)).
- Challenges of remote provision (compared to in-person support) for participants included reduced interaction/relationship building, issues with technology, and less accountability to complete work (Mahoney et al., 2021). The service provider's ability and level of comfort around the use of technology/remote provision was important in service user experience and perceptions of accessibility (Vera San Juan et al., 2021). The ability of providers to troubleshoot support issues, and clear guides around how to access/engage with remote sessions was noted as important (Vera San Juan et al., 2021).

Service provider perspectives

There was little work looking at provider/practitioner perspectives, but the existing work, combined with the qualitative work exploring service users' perspectives, highlights overall general positive perspectives of remote provision (Bennett et al., 2016; Appleton et al., 2021; Kim et al., 2018).

For smoking cessation staff, important trade-offs between remote and face-to-face approaches (Appleton et al., 2021) were noted. Whilst remote provision was described as more flexible, more efficient and convenient (Vera San Juan et al., 2020; Mahoney et al., 2021), and being more 'cost effective' (in terms of staff time), it was also seen as impacting upon rapport building, reducing the development of therapeutic relationships (Appleton et al., 2021; Vera San Juan et al., 2020; Mahoney et al., 2021). There were contradictory

findings around remote provision reducing engagement levels, as well as reducing the number of missed appointments, suggesting greater convenience may be improving accessibility (Appleton et al., 2021). Thus, this may be dependent upon different populations/individuals.

Within remote approaches, video calls were seen to offer more ‘personal’ and ‘interactive’ experiences than voice calls (Mahoney et al., 2021), resulting in higher rates of service user engagement/retention (Kim et al., 2018). Video calls, compared to voice calls, were also seen to enable service providers to identify and respond to (verbal and non-verbal) cues.

Potential learning

- Remote smoking cessation provision may provide an alternative acceptable and feasible treatment delivery modality, which has benefits in terms of access, costs and convenience for both service providers and service users. However, there are potential issues/challenges for both service users and providers.
- Whilst rapport has been noted as being possible to develop using remote means only (Cruvinel et al., 2019), providers’ skills and confidence in delivering remote support is important (Vera San Juan et al., 2020).
- The success of remote and online provision may be enhanced by the development of rapport through an initial (i.e., the first session) or ongoing face-to-face meetings (Mahoney et al., 2021; Vera San Juan et al., 2021).
- Remote approaches may be a beneficial way to deliver some kinds of support (e.g., brief interventions and check-ins (Vera San Juan et al., 2020; Appleton et al., 2021)), with more complex support being provided in-person.
- It appears that cessation rates are higher for those with higher rates of service engagement (e.g., attending more sessions (Ashton et al., 2015; Ormston et al., 2015; Zwar et al., 2015)). Therefore, being able to offer a range of approaches to best suit and engage service users appears crucial.
- Modality of care should be discussed on an individual basis, to find the best fit for service users’ preferences and circumstances. This requires flexible and personalised hybrid models of care.

3 Evaluability Assessment process

We used evaluability assessment methods to develop the evaluation design (Leviton, Khan et al. 2010, Craig and Campbell 2015). Evaluability assessment (EA) is a rapid, systematic, and collaborative way of deciding whether and how a programme or policy can be evaluated, and at what potential cost. We conducted three workshops with LWSF stakeholders to ascertain their understanding of how the LWSF hybrid model will deliver a better service for people wishing to quit smoking.

Workshop participants included people with lived experience of using employment services, local government, NHS and voluntary sector stakeholders, and observers from the Department of Work and Pensions and the Scottish Government. We allowed the workshop format to evolve to take account of feedback from preceding workshops, and to enable stakeholders to shape the approach to evaluation.

Table 1 Summary of workshop dates & agenda

**Workshop Number**	**Date**	**Agenda**
1	2 December 2021	Theory of Change development; understanding LWSF Hybrid during the pandemic
2	15 March 2022	Theory of Change sign-off; Evaluation aim & approach
3	17 May 2022	Evaluation questions & requirements

Workshop 1

The first LWSF EA workshop aimed to provide the evaluation team with an overview of the LWSF programme and aim, how LWSF was expected to bring about change, and what success would look like. A Theory of Change approach was used to clarify the intervention aims and desired outcomes. LWSF stakeholders shared their experiences of delivering a LWSF hybrid model during the Covid-19 lockdowns. Main themes discussed include:

- Rates
- Inequalities
- Wider health implications
- Accessibility/flexibility
- How it might influence other services

In particular, it was observed that LWSF has done well to adapt in this difficult time. There is an urgent need to try and understand the (changed) needs of service users and follow evidence best as we can. It was also noted that the service monitoring guidance is out of date (last updated in 2014). The hope is this EA process can help to update the guidance

Workshop 2

The second EA workshop focused on getting the Theory of Change signed off (Figure 1), as well as considering evaluation questions and data issues (like sources and collection methods) in relation to the evaluation design. This was considered from both the service

user as well as the service provider perspectives. Delivery and Service User-level outcomes were developed.

Workshop 3

The final EA workshop discussed a recalibration of the evaluation. There was now agreement that a feasibility study was preferred in the light of ongoing uncertainties around the delivery of LWSF. With a change in focus, stakeholders agreed evaluation questions should be framed around different aspects of the feasibility of the LWSF hybrid model.

Theory of Change

The LWSF Theory of Change (ToC) was co-developed with workshops participants and signed off in workshop 2. See Figure 1 below.

Figure 1 Living Well Smokefree Theory of Change

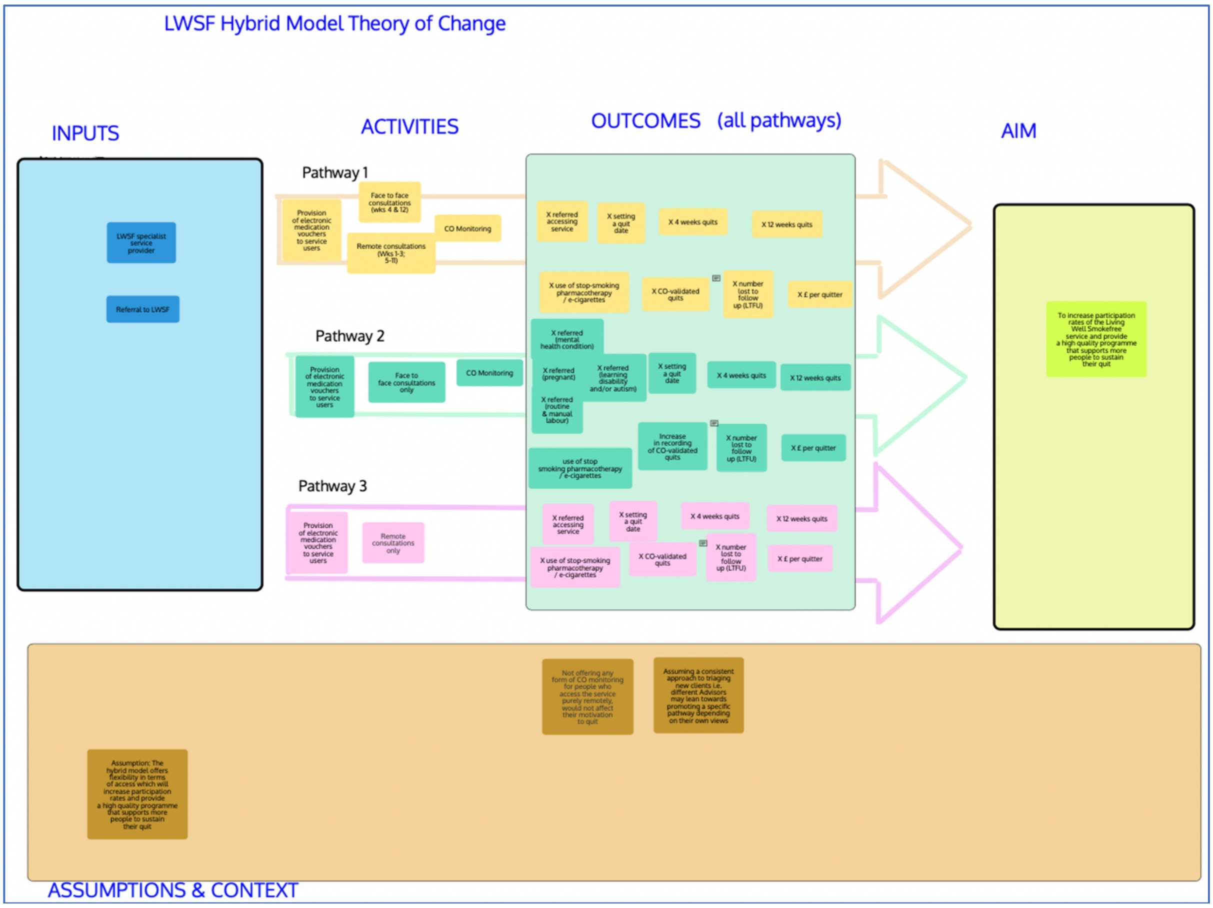

4 Evaluation design considerations

At the end of the EA workshops, LWSF stakeholders, together with PHIRST team members reached an understanding that the LWSF evaluation is a formative feasibility study. This is in line with current MRC guidance advocating the need to better understand the feasibility of the intervention design around optimal and acceptable intervention content and delivery; adherence to the intervention; likelihood of cost-effectiveness; and capacity of those providing the intervention to deliver as planned (Skivington, Matthews et al. 2021).

Aim & evaluation questions

The overall aim of the North Yorkshire County Council LWSF evaluation is to assess the feasibility of its implementation. The evaluation questions are:

1. What is the acceptability of LWSF to participants (non-target groups)?
   - What is the drop-off/LTFU (Lost To Follow Up)?
   - What is the reach and uptake?
   - What are the facilitators and barriers to participating in LWSF?
   - What are the experiences & perspectives of participants regarding face-to-face/blended/remote smoking cessation provision approaches?
2. What is the acceptability of LWSF to the target groups?
   - Which groups benefit the most/least?
   - What is the drop-off/LTFU (Lost To Follow Up)?
   - What is the reach and uptake within the target groups?
   - What are the facilitators and barriers to participating in LWSF?
   - What are the experiences & perspectives of participants regarding face-to-face/blended/remote smoking cessation provision approaches?
3. What is the acceptability of LWSF to delivery staff?
   - What are the facilitators and barriers to delivering LWSF?
   - What are the experiences & perspectives of service providers regarding face-to-face/blended/remote smoking cessation provision approaches?
   - Who are seen as key priority groups?
4. To what extent does existing monitoring & evaluation support understanding of how LWSF would reach and be effective for target groups?
   - What is the rationale/ underpinning assumptions behind the application of different modalities for different groups?
   - How is service provided/offered to service users
5. What can be learnt at mid-point (formative design)?
   - What are the strengths/weaknesses of LWSF for each pathway?
   - How can LWSF be adapted and improved?

Data

Data is currently collected on the QuitManager system and meets the reporting requirements of NHS Digital. To inform the LWSF KPIs, further investigation of service outputs and quitting outcomes is done by exporting data to Excel and cross-tabulating by client characteristics, source of referral, pharmacotherapy use etc. The LWSF service has an online dashboard that presents this information graphically and tabularly based on an established service performance framework.

5 Evaluation options

Four options are presented in this chapter for starting with the cheapest and most basic, and building on additional evaluation activity. See Table 1 for an overview:

- Option 0 involves no change or additional evaluation approach and relies on relevant and existing Monitoring & Evaluation (M&E) systems. We include Option 0 as a reference option. As discussed in the workshops, existing M&E systems do not address meet LWSF evaluation requirements.
- Option 1 supplements Option 0 with a summative process evaluation. A mixed method summative process evaluation of intervention and data collection system can provide end of evaluation findings for programme learning & adaptation.
- Option 2 is similar to Option 1 but is a formative evaluation approach with one check-in time point around the mid-point of the evaluation to provide timely data for programme learning & adaptation.
- Option 3 is similar to Option 2 but has more (quarterly) check-in time points to maximise opportunities for programme learning & adaptation.

Table 2 Overview of LWSF evaluation options

Evaluation Questions	Evaluation Design	Elaboration & Data Collection Tools	Pros	Cons
1. What is the acceptability of LWSF to participants (non-target groups)?<br>2. What is the acceptability of LWSF to the target groups?<br>3. What is the acceptability of LWSF to delivery staff?<br>4. To what extent does existing monitoring & evaluation support understanding of how LWSF would reach and be effective for target groups?<br>5. What can be learnt at mid-point (formative design)?	**Option 0 (reference option)**<br>Existing Monitoring & Evaluation (M&E)	• Routinely collected data for NHS Digital requirements	• No additional cost or resources required	• Unable to provide more timely data for programme learning & adaptation
	**Option 1**<br>Summative Process Evaluation	• Mixed method summative process evaluation of intervention & data collection system	• Can provide end of evaluation findings for programme learning & adaptation	• Unable to provide more timely data for programme learning & adaptation
	**Option 2**<br>Formative Process Evaluation (Mid)	• Mixed method formative process evaluation of intervention & data collection system with a mid-point check-in	• Can provide timely data for programme learning & adaptation	• Data for programme learning & adaptation limited to one time point
	**Option 3**<br>Formative Process Evaluation (Quarterly)	• Mixed method formative process evaluation of intervention & data collection system with quarterly check-ins	• Can provide more regular & timely data for programme learning & adaptation	• Most resource-intensive

The rest of this chapter describes each option in detail.

Option 0: Existing M&E

This is the reference option where no additional evaluation is done. This option instead relies on routinely collected data for NHS Digital requirements but will not support the aims of the LWSF evaluation which is met by the other options. We include Option 0 as a reference option. As discussed in the workshops, existing M&E systems do not address meet LWSF evaluation requirements.

Option 1: Summative Process Evaluation

The quantitative and qualitative data are designed to inform how to increase participation rates in the LWSF service and how to refine the service to increase the likelihood of quitting success. Both types of data collection will therefore have an emphasis on understanding variation in service use, experiences and outcomes among priority client groups. The qualitative data collection will go further to understand staff perspectives on the new hybrid service offering. The perspectives of staff will help to understand the facilitators and barriers to staff offering an efficient service to different client groups.

Quantitative investigation

The overall aim of quantitative data collection and use in the evaluation is to describe the demand for the different modes of contact offered. Descriptive statistics will be produced that show how demand and quitting outcomes differs among routes of referral and priority client groups.

Existing data collection and uses

Data is currently collected on the QuitManager system and meets the reporting requirements of NHS Digital. To inform the LWSF KPIs, further investigation of service outputs and quitting outcomes is done by exporting data to Excel and cross-tabulating by client characteristics, source of referral, pharmacotherapy use etc. The LWSF service has an online dashboard that presents this information graphically and tabularly based on an established service performance framework.

Setting

The data collected refers to clients seen in the community setting only (excluding clients seen in pharmacy or GP settings). *How does the hybrid model of LWSF fit alongside the options for getting support to stop smoking from the GP or pharmacy – do these also offer in-person and phone options?*

Addition of a new mode of contact field

Aims: To assign clients to one of three categories that then be used to stratify the existing tables of service use and outcomes. These reflect the three pathways defined in the logic models during the EA workshops.

Pathway 1: Clients who chose a hybrid option to begin with, or clients who chose to switch to a hybrid option later by starting with phone consultations only and then requesting an in-person consultation, or vice versa. Clients would be provided with an electronic medication voucher. Clients would then have a flexible mix of in person and phone consultations with smoking advisors. If CO monitoring is introduced into the service, then the timing of in-

person consultations could be planned to fit with the key points for CO monitoring. *If a client is on a phone only or in-person only option and some unplanned life event means that they need to temporarily switch to the other mode of contact, will they be classed as having a hybrid service?*

Pathway 2: Clients who chose in-person consultations to begin with and then stayed with in-person consultations only. Clients would be provided with an electronic medication voucher.

Pathway 3: Clients who chose phone consultations to begin with and then stayed with phone consultations only. Clients would be provided with an electronic medication voucher. *If a client is on a phone only option and they come for an in-person consultation to have a CO check, will they be classed as having a hybrid service? Will the service move to more single use CO monitors for remote clients to allow them to do their own CO monitoring?*

One suggestion is that a client should only be recorded as having switched to a hybrid mode of contact if they formally choose the hybrid option as part of a conversation with their advisor, e.g., on the basis of the hybrid option being a better option for their smoking cessation support.

Table 3 Important quantitative data fields

Item	Description	Options / decisions / questions
Mode of contact with the service	Mode of contact – there is a pre-existing field in QuitManager ("service provided") that records whether each client contact was face-to-face or remote. This field will not be used for service evaluation. Instead, a new field will be added with staff training on how to use it with the new service.	Proposed edits to QuitManager to create a new way of recording mode of contact:<br>At the first consultation with the client [on referral], the client will be asked to choose one of three options as their preference: Phone only, In person only, hybrid.<br>On subsequent consultations, a client who has previously chosen either phone or in-person only might then choose the hybrid option. Their original choice will remain in the system and the 'hybrid' option will also be ticked.<br>The LWSF team are currently exploring the cost and feasibility of two alternative options of implementing this with the QUITmanager team.
Number of referrals<br>Number accessed service<br>Number unable to contact<br>Number declined service	Numbers and percentages<br>Same as existing reporting	Use existing specifications within the QuitManager system<br>Expect that the client's preferred mode of contact under the new service is not yet known at the point in the service pathway where these data fields are recorded<br>These data fields would therefore not be stratified by mode of contact

		Important to understand whether the 'hybrid model' improves the numbers of people participating in the service i.e. not just referral numbers but the number of people who go on to access the service following triage (at present a large proportion of referred clients do not go on to start their programme).<br><br>Does the new hybrid offer improve the number of referrals that go on to start their quit journey?<br><br>*Is the client's preference for mode of contact (phone, in person, hybrid) expressed at the point of referral and before the 1st appointment?*
Number of clients had 1st appointment	The first consultation with a tobacco treatment advisor. This could be by phone or in person.	Split by the client's first choice mode of contact (phone, in person, hybrid)<br>Expect that the mode of contact of the first appointment will be the same as the client's first choice preference.<br>If the client chooses a hybrid preference, then the data collected for the evaluation would not be able to say whether this 1st appointment was in person or by phone
Provision of electronic medication vouchers to service users	Identified from EA workshop logic model	What are options for getting data on this from the QuitManager system?<br><br>Need to know relative use of different forms of pharmacotherapy / e-cigarettes between pathway options?<br>- potentially important for calculating cost per quit differences between pathways if the option to do so is chosen
Number of quit dates set	Indicator that a client is making an attempt to quit smoking	Split by the client's first choice mode of contact (phone, in person, hybrid)
Number of renewed quit attempts	Indicator that the initial quit attempt failed and a new attempt was begun	Split by the client's first choice mode of contact (phone, in person, hybrid)
Loss to follow-up	Client no longer contactable. If quit date set, then quitting outcome unknown.	Split by the client's first choice mode of contact (phone, in person, hybrid)<br><br>Do the new hybrid pathways result in a reduction in LTFU compared to the current mode?
Quit outcomes	Percentage of quits among clients who set a quit date<br>4, 12, 52 weeks<br>CO validation – if part of service	Split by the client's first choice mode of contact (phone, in person, hybrid)
Client characteristics (priority groups)	- Age (under 18, 18-34, 35-44, 45-59, 60+)<br>- Gender<br>- Ethnicity<br>- *Country of origin [Ukraine / Poland]*<br>- Occupational status (priority group is routine and manual)	Use existing specifications within the QuitManager system<br><br>Is an area-based deprivation measure available e.g. Index of Multiple Deprivation?

	- *District (7 districts in North Yorkshire County Council)*<br>- Pregnancy status<br>- Exemption from prescription charges<br>- Smoking history (number of cigarettes per day, date last smoked, number of quit attempts in previous year, Fagerstrom score, time from waking to first smoking)<br>- Mental health (Possibly from referral forms from health professionals. Key conditions might be learning disability / autism)<br>- Substance misuse (Possibly from referral forms from health professionals)<br>- Long-term health conditions (especially COPD and anything related to respiratory health / asthma. Heart attacks / strokes)	
Source of referral	Hospital<br>Emergency service<br>External stop smoking advisor<br>Dentist<br>Self-referral - *majority*<br>Maternity<br>HCP horizons / other<br>Primary care - specific GPs and pharmacies do deliver their own services - refer to LWSF if over capacity<br><br>*Also potentially relevant - "How heard" about the service*	

Qualitative investigation

The overall aim of the qualitative data collection is to understand staff and service users' perspectives on the service's offering of different modes of contact. This will provide data to inform refinements to how the service is implemented. In particular, the service is interested in how a hybrid mode of contact might enable them to tailor the service offering to priority groups, e.g., to achieve a better reach and better outcomes.

Timing

Service setup: The main activities will be focus groups with staff. This will further develop a theory of change for the new service. Staff perspectives on the service will be anonymised and summarised thematically to be presented back to service staff to support reflection service implementation so far.

Service implementation: This will involve further focus groups with staff to understand how perspectives change through the service implementation. It will also involve interviews with service users to understand their perspectives on the different modes of contact offered and how that affected their experience of the service.

Link between quantitative and qualitative data collection
Qualitative data collection will then aim to understand more details about client preferences for different modes of contact can be focused on sampling patients from each of the three categories of client above. Particular effort in qualitative data collection could be put into understanding the views of patients in the 'hybrid' category. This category will be a heterogenous mix of clients who chose hybrid initially, switched to hybrid and who had different mixes of phone and in-patient consultations.

Potential use of questionnaires
There is a pre-existing feedback form that is given to clients when they complete the service. However, few forms are returned. Contact with clients is made mainly by email. The service is considering a phone text option, and a financial incentive (voucher) to complete the feedback. The service is interested in using the evaluation process to understand how to their method of gaining feedback. The qualitative interviews with clients could look to understand the facilitators and barriers to clients providing feedback in this way.

Potential questions:
- Would you recommend this service to your friend/family member/colleague?
- What are key factors that helped you quit?
- If you participate again, would you choose the same mode of contact?

Everyone has a final meeting with an advisor so might be easy to collect questionnaire data during that meeting.

Questionnaires might be given to people when they enter the service too – to help understand the characteristics of patients lost to follow-up.

A questionnaire could be an easy way for the evaluation to collect data from a wider range of staff and service users.

Analyse surveys quantitatively to assess a change in key responses from baseline?

Table 4 Qualitative data to be collected

Item	Description
We will collect primary data including qualitative data from focus groups with service staff and other key stakeholders (including providers, commissionaires, cessation support workers), and interviews with service users currently involved in the LWSF service.	- Focus groups with key service staff/stakeholders involved in the delivery of the LWSF service. The focus groups will explore: the facilitators and barriers to delivering LWSF; the experiences and perspectives of service providers regarding face-to-face/blended/remote smoking cessation provision approaches; perception of how the service meets the needs of different clients/ different priority groups; what works well/what could be improved; comparison to previous approaches;

	thoughts on future service provision.<br><br>- Interviews with service users (including priority risk groups and non-priority groups). The interviews will be undertaken with services users towards the end of their treatment, to look at their experiences of the LWSF service. The interviews will explore: experiences and perspectives of participants regarding face-to-face/blended/remote smoking cessation provision approaches; the facilitators and barriers to participating in LWSF programme; what works well/what could be improved; thoughts on future service provision.

Option 2: Formative Process Evaluation (Mid)

Option 2 is similar to Option 1 but with the inclusion of an interim report/presentation with LWSF project partners where emerging findings are discussed and initial learning shared to help shape continued development of the service.

Option 3: Formative Process Evaluation (Quarterly)

Option 3 is similar to Option 2 but for the number of interim reporting to be increased to every three months.

6 Recommendation

Option 2 is the preferred option because it will help to define and then refine the service as the evaluation progresses. Option 2 would provide enough time for substantial data collection before the interim workshop (about 6 months in), and between the interim workshop and the evaluation final report.

Time period of data to be used for the evaluation

Start: When the new QuitManager fields that describe the new hybrid service are in operation (possibly with some time for the service to 'bed in'). *Possibly use data from a period (3 months?) before the new service was implemented to get an impression of the impact of the new hybrid offer on overall service use?*
Interim stop: In enough time to process the data for an interim report that would feed into an interim workshop with the service team to reflect on service implementation so far.
Final stop: In enough time to produce the final evaluation report. Anticipate a 12 to 18 month evaluation.

Development and qualitative description of the theory of change

The theory of change developed during the EA workshops will be further developed. Focus groups and interviews with staff and service users will add a thematic understanding of the key factors affecting service reach to priority groups, staff experience, client experience and quitting outcomes.

Key themes are likely to be:

- Data systems setup
- Staff training
- Client experience and journey through the new hybrid model
- Issues encountered by staff
- Resource implications of changing the service pathway

Statistical descriptions of service outputs and outcomes

Descriptive summary statistics will be produced that show:

- Numbers and percentages of clients choosing each pathway option at their first consultation
- Numbers and percentages of clients who subsequently switch from phone only or in-person only to hybrid
- How these numbers are split by priority client groups

Reflective meeting on evaluation findings together with the service

It is important to fit the outputs of the evaluation to the key time points for service commissioning and planning. It is recommended to have a workshop half way through the evaluation into which the evaluation could feed outputs to support reflection on service implementation so far by the service team. The outcomes of this workshop might be plans for refinement of the service implementation.

Modelling and the calculation of cost per quit

To calculate a monthly cost per 4-week quit of the service, we will estimate the monthly service cost (with and without including the sunk costs) and divide this by the monthly number of 4-week quits recorded by the service. This method is designed to be based on the method already used to compute the cost per quit of the service. We will not attempt to compute the cost per quit separately for each of the three pathways of the new hybrid service.

Public involvement and engagement

Involving past clients in advising on how to best collect data from current clients

Planning for the qualitative data collection from clients would benefit from advice from at least one past client on the best way to approach clients. This advice could be especially important for understanding how to best approach clients from certain priority groups e.g., with long term health conditions. The evaluation and service teams should discuss this further.

Involvement and engagement of LWSF staff in the evaluation

Service implementation and data recording

LWSF staff will need to be engaged from the beginning in understanding the new setup of the service and how to use the new features of the QUITManager recording system. LWSF staff will also need training to understand when a client should be marked as having had the hybrid service vs. another reason why a client might temporarily have a different mode of contact. There will need to be a consistent approach to triaging new clients (different advisors may lean towards promoting a specific pathway depending on their own views). Service staff need a clear briefing as to what the new service is and how to administer it and record service activity on QUITmanager. This training should happen at the start of the new service – in the subsequent qualitative data collection among staff, the usefulness of this training might be referred back to in relation to the realities of delivering the service.

Readiness to reflect on and adapt service implementation

LWSF staff engagement is particularly important for Option 2. The success of option 2 relies on LWSF staff being engaged in the service and being ready to reflect on findings and having the capacity to adapt.

Supporting the qualitative investigation

LWSF staff understanding of and involvement in the evaluation will be essential to help encourage them to participate in the evaluation by giving their own views in focus groups, and to gain their support in accessing other people in the organisation to participate in the staff-focused qualitative investigation. Engagement of smoking cessation advisors will further help to recruit service users to participate in interviews for the evaluation. Ideally, the advisors would know enough to be able to explain to clients why their involvement is important. This could be done by holding a meeting with staff at the start of service implementation.

Supporting the quantitative investigation – data processing and flows to ScHARR

Service outputs and quitting outcomes could be produced in the same way as the Excel reports on the service are already generated (pulling a report from QuitManager and then filtering and summarising in Excel). There are two options:

- Descriptive statistics could be produced on the service side and then the aggregated data transferred to ScHARR, who would then just present the descriptive statistics alongside the qualitative findings in the evaluation reports. This would require the ScHARR evaluation team to work closely alongside a LWSF team member to co-design the statistics produced to fit into the evaluation
- Alternatively, a version of the data from QuitManager could be transferred to ScHARR. The data in these reports would have to be anonymised first to abide by the relevant information governance requirements. The advantage of this second option is that ScHARR would then potentially have more flexibility to explore patterns in the data and tailor the presentation of findings to support the evaluation. This would also leave open the possibility of a more detailed / controlled statistical analysis of service effects.

References

Alqahtani, J.S., Aldhahir, A.M., Oyelade, T., Alghamdi, S.M. and Almamary, A.S., 2021. Smoking cessation during COVID-19: the top to-do list. NPJ Primary Care Respiratory Medicine, 31(1), pp.1-3.

Amos, A., Bauld, L., Hill, S., Platt, S. and Robinson, J., 2011. Tobacco control, inequalities in health and action at the local level in England. London: Public Health Research Consortium.

Appleton, R., Williams, J., San Juan, N.V., Needle, J.J., Schlief, M., Jordan, H., Rains, L.S., Goulding, L., Badhan, M., Roxburgh, E. and Barnett, P., 2021. Implementation, Adoption, and Perceptions of Telemental Health During the COVID-19 Pandemic: Systematic Review. Journal of medical Internet research, 23(12), p.e31746.

ASH. 2021a. Tobacco control and stop smoking services in local authorities in England, 2021. https://ash.org.uk/wp-content/uploads/2022/01/Reaching-Out.pdf

ASH. 2021b. Stepping up: The response of stop smoking services in England to the COVID-19 pandemic. https://ash.org.uk/wp-content/uploads/2021/01/ASH-CRUK-Stepping-Up-FINAL.pdf

ASH. 2022. Tobacco control and stop smoking services in local authorities in England, 2021. Reaching-Out.pdf (ash.org.uk)

Ashton, M., Rigby, A. and Galletly, C., 2015. Evaluation of a community-based smoking cessation programme for people with severe mental illness. Tobacco control, 24(3), pp.275-280.

Bennett, L., Jones, A. and Paranjothy, S., 2016. Models for Access to Maternal Smoking Cessation Support: a quasi-experiment to increase the engagement of pregnant women who smoke in National Health Service stop smoking services. The Lancet, 388, p.S23.

Braun, V. and Clarke, V., 2006. Using thematic analysis in psychology. Qualitative research in psychology, 3(2), pp.77-101.

Byaruhanga, J., Paul, C.L., Wiggers, J., Byrnes, E., Mitchell, A., Lecathelinais, C. and Tzelepis, F., 2020. Connectivity of real-time video counselling versus telephone counselling for smoking cessation in rural and remote areas: an exploratory study. International Journal of Environmental Research and Public Health, 17(8), p.2891.

Carlson, L.E., Lounsberry, J.J., Maciejewski, O., Wright, K., Collacutt, V. and Taenzer, P., 2012. Telehealth-delivered group smoking cessation for rural and urban participants: Feasibility and cessation rates. Addictive behaviors, 37(1), pp.108-114.

Celio, M.A., Mastroleo, N.R., DiGuiseppi, G., Barnett, N.P., Colby, S.M., Kahler, C.W., Operario, D., Suffoletto, B. and Monti, P.M., 2017. Using video conferencing to deliver a

brief motivational intervention for alcohol and sex risk to emergency department patients: a proof-of-concept pilot study. Addiction research & theory, 25(4), pp.318-325.

Cheuvront, M. 1998. Health communication on the Internet: an effective channel for health behavior change?. Journal of health communication, 3(1), pp.71-79. 25

Connolly, S.L., Miller, C.J., Lindsay, J.A. and Bauer, M.S., 2020. A systematic review of providers' attitudes toward telemental health via videoconferencing. Clinical Psychology: Science and Practice, 27(2), p.e12311

Craig, P. and M. Campbell (2015). "Evaluability Assessment: a systematic approach to deciding whether and how to evaluate programmes and policies."

Crane, D., Ubhi, H.K., Brown, J. and West, R., 2018. Relative effectiveness of a full versus reduced version of the 'Smoke Free' mobile application for smoking cessation: An exploratory randomised controlled trial. F1000Research, 7.

Danielsson, A.-K.; Eriksson, A.-K.; Allebeck, P. Technology-based support via telephone or web: A systematic review of the effects on smoking, alcohol use and gambling. Addict. Behav. 2014, 39, 1846–1868

Di Cesare M, Khang Y-H, Asaria P, Blakely T, Cowan MJ, Farzadfar F, et al. Inequalities in non-communicable diseases and effective responses. Lancet. 2013;381(9866):585–97.

Ekeland AG, Bowes A, Flottorp S. 2010. Effectiveness of telemedicine: a systematic review of reviews. Int J Med Inform. Nov;79(11):736-771

Getty, C.A., Morande, A., Lynskey, M., Weaver, T. and Metrebian, N., 2019. Mobile telephone‐delivered contingency management interventions promoting behaviour change in individuals with substance use disorders: a meta‐analysis. Addiction, 114(11), pp.1915-1925.

Haluza, D., Saustingl, M. and Halavina, K., 2020. Perceptions of practitioners on telehealth and app use for smoking cessation and COPD care—An exploratory study. Medicina, 56(12), p.698.

Hilty, D.M., Ferrer, D.C., Parish, M.B., Johnston, B., Callahan, E.J. and Yellowlees, P.M., 2013. The effectiveness of telemental health: a 2013 review. Telemedicine and e-Health, 19(6), pp.444-454.

Hiscock, R., Judge, K. and Bauld, L., 2011. Social inequalities in quitting smoking: what factors mediate the relationship between socioeconomic position and smoking cessation?. Journal of public health, 33(1), pp.39-47.

Joyce, C.M., Saulsgiver, K., Mohanty, S., Bachireddy, C., Molfetta, C., Steffy, M., Yoder, A. and Buttenheim, A.M., 2021. Remote Patient Monitoring and Incentives to Support Smoking

Cessation Among Pregnant and Postpartum Medicaid Members: Three Randomized Controlled Pilot Studies. JMIR formative research, 5(9), p.e27801.

Kim, S.S., Darwish, S., Lee, S.A., Sprague, C. and DeMarco, R.F., 2018. A randomized controlled pilot trial of a smoking cessation intervention for US women living with HIV: telephone-based video call vs voice call. International journal of women's health, 10, p.545.

Kim, S.S., Sitthisongkram, S., Bernstein, K., Fang, H., Choi, W.S. and Ziedonis, D., 2016. A randomized controlled trial of a videoconferencing smoking cessation intervention for Korean American women: preliminary findings. International journal of women's health, 8, p.453.

Kooistra, L.C., Ruwaard, J., Wiersma, J.E., van Oppen, P., van der Vaart, R., van Gemert-Pijnen, J.E. and Riper, H., 2016. Development and initial evaluation of blended cognitive behavioural treatment for major depression in routine specialized mental health care. Internet interventions, 4, pp.61-71.
Leviton, L. C., L. K. Khan, D. Rog, N. Dawkins and D. Cotton (2010). "Evaluability assessment to improve public health policies, programs, and practices." Annu Rev Public Health **31**: 213-233.

Mahoney, M.C., Park, E., Schlienz, N.J., Duerr, C. and Hawk, L.W., 2021. Transitioning to remote clinic visits in a smoking cessation trial during the COVID-19 Pandemic: mixed methods evaluation. JMIR Formative Research, 5(4), p.e25541.

Mardle, T., Merrett, S., Wright, J., Percival, F. and Lockhart, I., 2012. Real world evaluation of three models of NHS smoking cessation service in England. BMC Research Notes, 5(1), pp.1-6.

Matkin W, Ordoñez-Mena JM, Hartmann-Boyce J. 2019. Telephone counselling for smoking cessation. Cochrane Database Syst Rev. 5. https://doi.org/10.1002/14651858.CD002850.pub4

Michael, M.C.C.J.B.C. and CHEUVRONT, C.C.J.B., 1998. Health communication on the Internet: an effective channel for health behavior change?. Journal of health communication, 3(1), pp.71-79.

NHS Digital. 2021. Statistics on Women's Smoking Status at Time of Delivery: England—Quarter 3-2020-21. 3 June 2021.https://digital.nhs.uk/data-and-information/publications/statistical/statistics-on-women-s-smoking-status-at-timeof-delivery-england/statistics-on-womens-smoking-status-at-time-of-delivery-england---quarter-3-2020-21

NHS. (2019) NHS long term plan. https://www.longtermplan.nhs.uk/online-version/chapter-2-more-nhs-action-on-prevention-and-health-inequalities/smoking/

Nomura, A., Tanigawa, T., Muto, T., Oga, T., Fukushima, Y., Kiyosue, A., Miyazaki, M., Hida, E. and Satake, K., 2019. Clinical efficacy of telemedicine compared to face-to-face clinic visits

for smoking cessation: multicenter open-label randomized controlled noninferiority trial. Journal of medical Internet research, 21(4), p.e13520.

North Yorkshire tobacco control strategy 2015-25. 2015. https://www.nypartnerships.org.uk/sites/default/files/Partnership%20files/Health%20and%20wellbeing/Winter%20health/Tobacco%20control%20strategy.pdf

Ormston, R., van der Pol, M., Ludbrook, A., McConville, S. and Amos, A., 2015. quit4u: the effectiveness of combining behavioural support, pharmacotherapy and financial incentives to support smoking cessation. Health Education Research, 30(1), pp.121-133.

School for Public Health Research, 2018. The 6 SPHR Knowledge Sharing Principles. https://sphr.nihr.ac.uk/wp-content/uploads/2018/10/Appendix-2_Knowledge-sharing-principles.pdf.

Siemer, L., Allouch, S.B., Pieterse, M.E., Brusse-Keizer, M., Sanderman, R. and Postel, M.G., 2020. Patients' User Experience of a Blended Face-to-Face and Web-Based Smoking Cessation Treatment: Qualitative Study. JMIR formative research, 4(6), p.e14550

Skivington, K., L. Matthews, S. A. Simpson, P. Craig, J. Baird, J. M. Blazeby, K. A. Boyd, N. Craig, D. P. French, E. McIntosh, M. Petticrew, J. Rycroft-Malone, M. White and L. Moore (2021). "A new framework for developing and evaluating complex interventions: update of Medical Research Council guidance." BMJ **374**: n2061.

Spanakis, P., Peckham, E., Young, B., Heron, P., Bailey, D. and Gilbody, S., 2021. A systematic review of behavioural smoking cessation interventions for people with severe mental ill health—what works?. Addiction.

Tall, J.A., Brew, B.K., Saurman, E. and Jones, T.C., 2015. Implementing an anti-smoking program in rural-remote communities: challenges and strategies. Rural and Remote Health, 15(4), pp.140-154.

Thomsen, T., Villebro, N. & Møller, A. M.2014. Interventions for preoperative smoking cessation. Cochrane Database Syst. Rev. 2014, Cd002294

Tzelepis, F., Paul, C.L., Williams, C.M., Gilligan, C., Regan, T., Daly, J., Hodder, R.K., Byrnes, E., Byaruhanga, J., McFadyen, T. and Wiggers, J., 2019. Real-time video counselling for smoking cessation. Cochrane Database of Systematic Reviews, (10).

Vera San Juan, N., Shah, P., Schlief, M., Appleton, R., Nyikavaranda, P., Birken, M., Foye, U., Lloyd-Evans, B., Morant, N., Needle, J.J. and Simpson, A., 2021. Service user experiences and views regarding telemental health during the COVID-19 pandemic: A co-produced framework analysis. PloS one, 16(9), p.e0257270.

Vinci, C., Hemenway, M., Baban, S.S., Yang, M.J., Brandon, K.O., Witkiewitz, K., Unrod, M., Brandon, T.H., Wetter, D.W. and Sutton, S.K., 2022. Transition to telehealth: Challenges and

benefits of conducting group-based smoking and alcohol treatment virtually. Contemporary Clinical Trials, p.106689.

West, R., 2005. Assessing smoking cessation performance in NHS stop smoking services: The Russell standard (clinical). London: National Centre for Smoking Cessation and Training.

(WHO) World Health Organization. 2017a. WHO report on the global tobacco epidemic, 2017: monitoring tobacco use and prevention policies. World Health Organization. https://apps.who.int/iris/handle/10665/255874.

(WHO) World Health Organization. 2017b. Tobacco Fact sheet. World Health Organization. 2017. http://www.who.int/mediacentre/factsheets/fs339/en/

Wilhelmsen, M., Lillevoll, K., Risør, M.B., Høifødt, R., Johansen, M.L., Eisemann, M. and Kolstrup, N., 2013. Motivation to persist with internet-based cognitive behavioural treatment using blended care: a qualitative study. BMC psychiatry, 13(1), pp.1-9.

Wu, F., Burt, J., Chowdhury, T., Fitzpatrick, R., Martin, G., Van Der Scheer, J.W. and Hurst, J.R., 2021. Specialty COPD care during COVID-19: patient and clinician perspectives on remote delivery. BMJ open respiratory research, 8(1), p.e000817.

Zwar, N.A., Richmond, R.L., Halcomb, E.J., Furler, J.S., Smith, J.P., Hermiz, O., Blackberry, I.D., Jayasinghe, U.W. and Borland, R., 2015. Quit in general practice: a cluster randomized trial of enhanced in-practice support for smoking cessation. Family practice, 32(2), pp.173-180.
