# Supplementary material for: Reintroducing face-to-face support alongside remote support to form a hybrid stop smoking service in England: a formative mixed methods evaluation
Source: BMC Public Health. 2024 Mar 6;24:718. doi: 10.1186/s12889-024-18235-0 (PMC10916048; doi:10.1186/s12889-024-18235-0)
Supplement: Supplementary file 2 — Supplementary Material 2 [file 12889_2024_18235_MOESM2_ESM.docx]

**Supplementary material for “Reintroducing face-to-face support alongside remote support to form a hybrid stop smoking service in England: A formative mixed methods evaluation”**

Additional file 2

**Information Sheets and Consent forms**

1. Stakeholder information sheet  (page 2)
2. Service user information sheet (page 7)
3. Stakeholder consent form (page 12)
4. Service user consent form (page 14)
5. Stakeholder topic guide (page 17)
6. Service user topic guide (page 19)
7. Coding framework (page 21)
8. Service user demographic information (page 23)


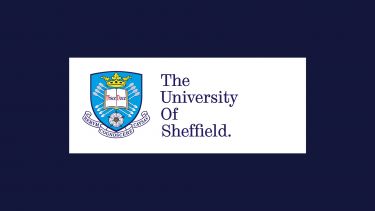


**Stakeholder information sheet**

You are being invited to take part in a research project called, ‘**Evaluation of the Living Well Smokefree Hybrid Specialist Stop Smoking Service**’. To help you decide whether or not to participate, it is important for you to understand why the research is being done and what it will involve. Please read the following information carefully and take time to decide whether or not you wish to take part in the study. The research team are happy to answer any questions you may have about the study, and contact information is provided at the end of this information sheet.

| **Purpose of the study** |
| --- |

Community stop smoking services are at the forefront of efforts to reduce premature death and disability due to smoking in the UK. There are approximately 70,000 smokers within North Yorkshire, with higher rates being found in areas of greater deprivation, and worryingly, above national average rates of smoking during pregnancy.

Due to the Covid-19 pandemic, to continue to support individuals wishing to quit smoking, the North Yorkshire Living Well Smokefree (LWSF) service moved from face-to-face provision, to remote delivery with support being delivered in virtual formats (voice/video call). As Covid-19 restrictions have eased, LWSF have begun to develop a ‘hybrid’ approach, which includes three service delivery modalities, where smoking cessation support is offered either face-to-face, remotely (via voice/video calls), or through a ‘blended’ approach (a combination of face-to-face and remote approaches).

This study seeks to evaluate the feasibility and acceptability of this hybrid smoking cessation service, to help LWSF develop an effective model for service users.

The study is led by PHIRST Fusion, and is funded by the National Institute for Health Research (NIHR).

The PHIRST Fusion team is led from Fuse (the Centre for Translational Research in Public Health in North East England) in collaboration with PaRC (the Public Health Practice and Research Collaborative for Yorkshire & Humber); the Centre of Excellence for Public Health in Northern Ireland; the Scottish Collaboration for Public Health Research and Policy and the MRC/CSO Social & Public Health Sciences Unit.

PHIRST Fusion links up academic teams with local authorities to evaluate public health schemes and work.

| **Why am I being invited to participate?** |
| --- |

You are being asked to take in a focus group as we want to speak to people involved in the delivery and design of the ‘hybrid’ LWSF service.

There is a need to look at if different provision approaches are impacting upon smoking cessation (including quit rates, engagement levels), and if hybrid and blended approaches are effective, feasible and acceptable delivery models. We need to speak to people directly involved with the LWSF service to hear their thoughts and to listen to their ways to improve the service for staff and service users.

Taking part is completely up to you.

| **What does taking part involve**? |
| --- |

Taking part involves participating in a focus group or group interview with a member of the research team and [1. (for LWSF staff who deliver the LWSF programme) other smoking cessation advisers only] [2. (for people involved in the development of the LWSF service) other LWSF service managers/commissioners] discussing your perceptions of the LWSF service and its hybrid approach.

The focus group will take place face to face or online during your LWSF team meeting time on [*add date*]. With your permission the focus group will be audio recorded.

The focus group will last approximately 120 minutes.

We will repeat this focus group in several months to see if your thoughts have changed.

You will be asked to complete and sign a consent form to take part.

| **What will the focus group be about?** |
| --- |

The focus group will explore your perspectives around the LWSF hybrid model, and experiences of working with the programme. In the focus group we will talk about:

- the facilitators and barriers to delivering LWSF hybrid model
- your experiences and perspectives of face-to-face/blended/remote smoking cessation provision
- your perceptions of how the service meets the needs of different clients/ different priority groups
- what works well/what could be improved
- your thoughts on future service provision

| **Do I have to take part?** |
| --- |

Taking part is entirely up to you. You do not have to take part if you do not want to.

If you do take part you will be invited to a focus group with LWSF staff members and members of the research team.

We will ask you to complete a consent form before participating to make sure you are happy to take part, that you understand what taking part involves, and you know what we will do with the information you give us.

If you change your mind, you can stop taking part at any time without giving a reason, this includes during the focus group session.

If you do want to withdraw from the study, any personal details we have about you will be deleted.

Because of the nature of focus groups and the audio recording of the session, it is not possible to withdraw participation after the focus group due to difficulties in identifying individual participants. So please carefully consider your participation before deciding to take part. Again, please contact the research team to discuss - contact information is presented at the end of this information sheet.

If you want to take part please inform [LWSF service manager] as soon as possible.

| **Are there any possible benefits or downsides to taking part?** |
| --- |

While there are no immediate benefits for participants, we hope that you will enjoy talking about your perspectives of the hybrid LWSF programme, and enjoy having the time and space to talk about how you think the service could be made better.

If you feel uncomfortable in any way or decide you no longer want to take part, then you can stop at any time without giving a reason. Also, you can skip questions and you do not have to answer a question if you do not want to.
We would encourage that do not share any thoughts around the LWSF service that you wouldn’t want other people or your colleagues to know about. Please carefully consider your participation before deciding to take part.

We believe the main disadvantage of participating is the time this will take out of your working day. However, the focus group will be run as part of your team meeting time.

| **Is taking part confidential?** |
| --- |

The research team will keep what is said in the focus groups confidential. The only time we may have to tell someone what you have said is if you say something which suggests there is a risk of significant harm to someone (including you). If this happens we will talk about what will happen next.

Any information we use in our research (e.g., quotes) will be anonymised (i.e. your name or anything else that could identify you will not be used).

We will encourage everyone taking part in the focus groups to keep what they hear in the focus groups private. However, we cannot guarantee that other people in the focus group will keep what is said in the focus group private. So we suggest that you do not share anything that you wouldn’t want other people to know about.

| **What will happen to the data collected?** |
| --- |

Any personal information we collect about you during the research will be kept strictly confidential and will only be accessible to members of the research team. We may have to record some personal details about you to help you take part (e.g., your name, work email address), but this will be securely stored in password protected spreadsheets on a secure online folder, and these details will be deleted as soon as possible after your focus group.

The focus group will be audio recorded using an encrypted digital recorder. Afterwards the conversation will be typed up on a computer with all personal information (e.g., names) removed. All copies of the recording will be destroyed one month after being typed-up. Transcripts will be anonymised, securely stored on the University of Sheffield network drives and accessible only to the project team for the duration of the project. After the project the anonymised transcripts will be deposited in a secure online data repository at Sheffield University (called ORDA) and may be made available for analysis by other researchers on request and the research team will ensure shared data are censored where necessary to minimise identifiability risks.

Consent forms will be scanned onto and stored securely in a restricted folder on the University of Sheffield's secure drive. Paper copies of consent forms will then be destroyed by shredding and confidential waste disposal. Consent forms will be securely stored for 10 years and then deleted.

| **Who is responsible for looking after my information?** |
| --- |

The University of Sheffield will act as Data Controller for this study. This means they are responsible for looking after your information and using it properly.

| **What is the legal basis for processing my personal data?** |
| --- |

Data protection laws say we have to explain to you why we are asking for the personal information we will collect in the focus groups. We need this information for research that is a task in the public interest – this means something that is good for the general public.

| **What will happen to the findings of the research?** |
| --- |

The results will be written up in reports and published. The results will be used to help shape and improve the LWSF service. The findings from the focus groups will be written up and fed back to service managers and commissioners.

While we intend to share the findings from this work in reports, articles and presentations, and whilst your words may be quoted in the publications and other research outputs but they will not be attributed to you nor will any information that may reveal your participation be published. Your name, role or other identifying details will not be published and you will not be identifiable in any reports or publication.

| **Ethical Review** |
| --- |

This project has been checked by the University of Sheffield Research Ethics Committee through the School of Health and Related Research.

| **What if I have any questions?** |
| --- |

If you have any questions or are unhappy about anything, please let Nick Woodrow or another member of the research team know (contact details are listed at the end of this information sheet). If you would prefer to talk to someone outside of the research team, you can contact Professor Mark Strong, Dean of the School of Health and Related Research,

If you have any issues about the way we handle your personal data, you can contact the University’s Data Protection Officer, Luke Thompson on 0114 2221117 or dataprotection@sheffield.ac.uk. If you are not satisfied with how your complaint is handled, you may then escalate the complaint to the ICO (Information Commissioner’s Office).

| **How can I find out more about this project?** |
| --- |

If you would like to ask any questions about this project, you can contact:

University of Sheffield:

Nick Woodrow at [n.woodrow@sheffield.ac.uk](mailto:n.woodrow@sheffield.ac.uk)

Liddy Goyder at [e.goyder@sheffield.ac.uk](mailto:e.goyder@sheffield.ac.uk)

Duncan Gillespie at [duncan.gillespie@sheffield.ac.uk](mailto:duncan.gillespie@sheffield.ac.uk)

Thank you very much for reading this!


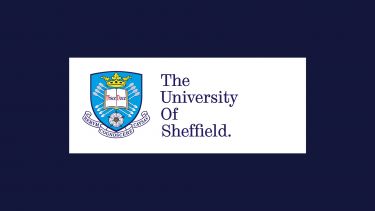


**Service user information sheet**

You are being invited to take part in a research project called, ‘**Evaluation of the Living Well Smokefree Hybrid Specialist Stop Smoking Service**’. To help you decide whether or not to participate, it is important for you to understand why the research is being done and what it will involve. Please read the following information carefully and take time to decide whether or not you wish to take part in the study. The research team are happy to answer any questions you may have about the study, and contact information is provided at the end of this information sheet.

| **Key points** |
| --- |
| - You are being invited to take part in an interview which will last around 30 minutes - The interview is about what you think about your smoking cessation support, and ways it could be made better - it’s not about your current smoking - You will get a £15 shopping voucher for taking part in the interview as a thank you for your time - What you say in the interview will be kept confidential, that means we will not tell anyone what you have said. The only time we may have to tell someone what you have said is if you say something which suggests there is a risk of significant harm to someone (including you) - You do not have to take part if you do not want to, and can change your mind about taking part at any point - Taking part in the research will have no impact on your treatment |

| **Why are we doing this research** |
| --- |

Community stop smoking services are at the forefront of efforts to reduce smoking in the UK.

Due to the Covid-19 pandemic, to continue to support individuals wishing to quit smoking, the North Yorkshire Living Well Smokefree (LWSF) service moved from face-to-face provision, to remote delivery with support being delivered in virtual formats (voice/video call). As Covid-19 restrictions have eased, LWSF have begun to develop an approach of delivering smoking cessation support in three different ways: 1) face-to-face, 2) remotely (via voice/video calls), 3) through a ‘blended’ approach (a combination of face-to-face and remote approaches).

This study is looking at how useful these ways of delivering support are, and we are wanting to speak to people using the LWSF service to see what they think about it, and if they have any ideas on how to make the service better.

The study is led by PHIRST Fusion, and is funded by the National Institute for Health Research (NIHR).

The PHIRST Fusion team is led from Fuse (the Centre for Translational Research in Public Health in North East England) in collaboration with PaRC (the Public Health Practice and Research Collaborative for Yorkshire & Humber); the Centre of Excellence for Public Health in Northern Ireland; the Scottish Collaboration for Public Health Research and Policy and the MRC/CSO Social & Public Health Sciences Unit.

PHIRST Fusion links up academic teams with local authorities to evaluate public health schemes and work.

| **Do I have to take part?** |
| --- |

Taking part in the interview is entirely up to you. You do not have to take part if you do not want to.

If you do take part you will be invited to an interview with someone from our research team. This interview will take place face-to-face, over the phone, or through an online video call - we will arrange whatever you prefer.

With your permission, the interview will be audio recorded as it is important that we remember what you say to us. If the interview is on an online video call, it will be audio recorded only, there is no video recording.

If you are interested in taking part, let your LWSF smoking cessation adviser know and they will pass the research team your contact information. Someone from the research team will be in contact to discuss the research, and everything will be organised for you.

The interview will last about 30 minutes.

We will ask you to complete a consent form before participating to make sure you are happy to take part, and that you understand what taking part involves.

You will get a £15 shopping voucher for taking part in the interview as a thank you.

If you change your mind about taking part, that is okay. You do not have to take part if you do not want to. If you change your mind, you can withdraw from the study at any time without giving a reason. This includes after the interview has taken place. To withdraw from the study you should contact one of the research team (contact details are at the end of this information sheet). If the interview has taken place, you can withdraw from the study up to one week after completion of the interview, by contacting the research team and providing them with your participation number, which will be written on your consent form. After this date it will not be possible to withdraw your interview from the study, due to any personal information linking it to you being removed, but we will delete any personal details we have about you.

| **Are there any possible benefits or downsides to taking part?** |
| --- |

We hope that you will enjoy discussing your experiences and perspectives of the LWSF service with us and enjoy having the chance to put forward your thoughts and opinions about how to make the LWSF service better.

Taking part in the research will have no impact on your treatment.

We will not be asking questions around your personal situations and smoking practices. We will only ask you about your thoughts around the LWSF programme more generally. For example we make ask you about:

- What it’s like receiving support in person or over the phone
- What are the good things and bad things about this
- what works well/what could be improved
- your thoughts on future service provision and what you’d like to see changed

It is important to know that you do not have to answer any questions you do not want to. If you feel uncomfortable in any way or decide you no longer want to take part, then you can stop at any time without giving a reason. Also, you can skip questions and you do not have to answer a question if you do not want to.

| **Is taking part confidential?** |
| --- |

Any information we use in our research (e.g., any quotes) will be anonymised (i.e. your name or anything else that could identify you will not be used). The only time we may have to tell someone what you have said is if you say something which suggests there is a risk of significant harm to someone (including you). If this happens we will talk about what will happen next.

Any personal information we collect about you during the research will be kept strictly confidential and will only be accessible to members of the research team. We’ll have to record some personal details about you so we can set up the interview (e.g., your name, your contact information) but this will be stored in a password protected spreadsheet on the University of Sheffield's secure systems, and these details will be deleted as soon as possible after your interview.

All interviews will be audio recorded using a digital recorder. Afterwards your interview will be typed up on a computer with all personal information removed so you can’t be identified. All copies of the recording will be destroyed one month after being typed-up. Typed-up interviews will be kept securely in restricted folders on password protected computers that are only accessible by the research team. After the project we will also store the typed up interview securely online in a data repository called ORDA; a data repository is a place where research information can be kept after a project has finished for other people to use. This means that other researchers may read the typed up interviews in the future, but they will not have any way of linking this to you as all personal information will be removed.

Consent forms will be scanned onto and stored securely in a restricted folder on the University of Sheffield's system. Any paper copies of consent forms will then be destroyed by shredding and confidential waste disposal. Consent forms will be securely stored for 10 years and then deleted.

| **Who is responsible for looking after my information?** |
| --- |

The University of Sheffield will act as the Data Controller for this study. This means the University of Sheffield is responsible for looking after your information and using it properly.

| **What is the legal basis for processing my personal data?** |
| --- |

Data protection laws say we have to explain to you why we are asking for the personal information we will collect in the interviews and what we will do with it. We need this information for research that is a task in the public interest – this means something that is good for the general public.

| **What will happen to the findings of the research?** |
| --- |

We intend to share the findings from this work in reports, articles and presentations. The results will be written up so that the LWSF service can be improved. Therefore, we will write up what you tell us to help improve the LWSF service, but nothing will be linked back to you. Whilst your words may be quoted in the publications and other research outputs but they will not be attributed to you nor will any information that may reveal your participation be published.

| **Has anyone checked this project is okay?** |
| --- |

Before any research is allowed to happen it has to be checked by a group of people called an Ethics Committee. They make sure the research is okay to do. This project has been checked by the University of Sheffield Research Ethics Committee through the School of Health and Related Research.

| **What if I have any problems?** |
| --- |

If you have any questions or are unhappy about anything, please let Nick Woodrow or another member of the research team know (contact details are listed at the end of this information sheet). If you would prefer to talk to someone outside of the research team, you can contact Professor Mark Strong, Dean of the School of Health and Related Research.

If you have any issues about the way we handle your personal data, you can contact the University’s Data Protection Officer, Luke Thompson on 0114 2221117 or dataprotection@sheffield.ac.uk. If you are not satisfied with how your complaint is handled, you may then escalate the complaint to the ICO (Information Commissioner’s Office).

| **How can I find out more about this project?** |
| --- |

If you would like to ask any questions about this project, you can contact

University of Sheffield:

Nick Woodrow at [n.woodrow@sheffield.ac.uk](mailto:n.woodrow@sheffield.ac.uk)

Liddy Goyder at [e.goyder@sheffield.ac.uk](mailto:e.goyder@sheffield.ac.uk)

Duncan Gillespie at [duncan.gillespie@sheffield.ac.uk](mailto:duncan.gillespie@sheffield.ac.uk)

Thank you very much for reading this!

**Stakeholder
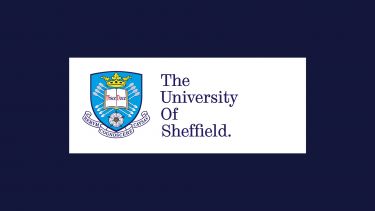
consent form**

**Evaluation of the Living Well Smokefree Hybrid Specialist Stop Smoking Service**

**Consent Form**

| **Participant number** |  |
| --- | --- |

| **Taking Part in the Project** | |  |
| --- | --- | --- |
|  | ***Please tick if you agree*** |  |
| I have read and understood the project information sheet, dated __________. (If you will answer ‘No’ to this question please do not proceed with this consent form until you are aware of what your participation in the project will mean). |  |  |
| I have been given the opportunity to ask questions about the project. |  |  |
| I understand that taking part in the project will include participating in a focus group and I consent to this being audio recorded. I understand there will be no video recording. |  |  |
| I understand that my taking part is voluntary,  and that I can stop at any time without giving a reason. |  |  |
| I understand that I can withdraw my personal details from the study by contacting the research team. |  |  |
| **How my information will be used during and after the project** | |  |
|  | ***Please tick if you agree*** |  |
| I understand any personal details (such as name, phone number, address and email address etc.) will not be shared with people outside the project. |  |  |
| I understand and agree that my words may be quoted in publications, reports, web pages, and other research outputs. I understand that I will not be directly named in these outputs. |  |  |
| I understand that the information from the focus groups may be used to support other research in the future, and may be shared anonymously with other researchers. |  |  |
| I give permission for the focus group data that I provide to be deposited in a secure online repository (ORDA) so it can be used for future research and learning. I understand that my name will not be included in any of the information which goes into the archive. |  |  |
| **So that the information you provide can be used legally by the researchers** | |  |
|  | ***Please tick if you agree*** |  |
| I agree to assign the copyright I hold in any materials generated as part of this project to The University of Sheffield. |  |  |

**______________________ ____________________ _____________**

**Name of participant [printed] Signature Date**

**________________________ ____________________ _____________**

**Name of researcher [printed] Signature Date**

**Service user consent form**


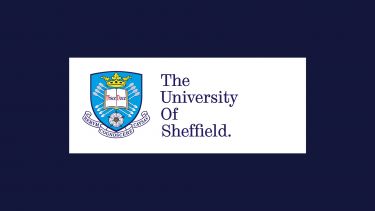


**Evaluation of the Living Well Smokefree Hybrid Specialist Stop Smoking Service**

**Consent Form**

| **Participant number** |  |
| --- | --- |

| **Taking Part in the Project** | |  |
| --- | --- | --- |
|  | ***Please tick if you agree*** |  |
| I have read and understood the project information sheet, dated __________. (If you will answer ‘No’ to this question please do not proceed with this consent form until you are aware of what your participation in the project will mean). |  |  |
| I have been given the opportunity to ask questions about the project. |  |  |
| I understand that taking part in the project will include participating in an interview and I consent to my interview being audio recorded. |  |  |
| I understand that my taking part is voluntary,  and that I can stop at any time without giving a reason. |  |  |
| I understand that I can withdraw my personal details from the study by contacting the research team and quoting the unique participant number on this form. |  |  |
| I understand I can withdraw my interview from the study at any time up to  ___________, which is one week after the interview date.  I understand that after this date my interview cannot be separated out and removed from the study. I understand that I can do this by contacting the research team and quoting the unique participant number on this form. |  |  |
| **How my information will be used during and after the project** | |  |
|  | ***Please tick if you agree*** |  |
| I understand my personal details (such as name, phone number, address and email address etc.) will not be shared with people outside the project. |  |  |
| I understand and agree that my words may be quoted in publications, reports, and other research outputs. I understand that I will not be directly named in these outputs. |  |  |
| I understand and agree that other authorised researchers will have access to this data only if they agree to preserve the confidentiality of the information as requested in this form. |  |  |
| I understand and agree that other authorised researchers may use my data in publications, reports, web pages, and other research outputs, only if they agree to preserve the confidentiality of the information as requested in this form. |  |  |
| I give permission for the interview data that I provide to be deposited in a secure online repository (ORDA) so it can be used for future research and learning. I understand that my name will not be included in any of the information which goes into the archive. |  |  |
| **So that the information you provide can be used legally by the researchers** | |  |
|  | ***Please tick if you agree*** |  |
| I agree to assign the copyright I hold in any materials generated as part of this project to The University of Sheffield. |  |  |

**______________________ ____________________ _____________**

**Name of participant [printed] Signature Date**

**________________________ ____________________ _____________**

**Name of researcher [printed] Signature Date**

**Stakeholder topic guide**

Introduction:

- Thank you for participating
- Introduction of self
- introduction of study
  - *basically we’re evaluating the hybrid model (where support is provided f2f, remotely, and blended using both) & wanting to speak to people involved to see what they think about it*
- Key points
- length of focus group (60 minutes)
- Focus group *so please discuss and respond amongst yourself*
- no right or wrong answers - exploring perspectives
- participation is voluntary
- confidentiality/anonymity = *don’t share what you wouldn’t want others to know*
- Focus group will be audio recorded
- Questions? Happy to proceed?
- Complete and sign consent form = *if all read I can fill this in*

*Participant role/background*

- Clarify current role and responsibilities
- Role in relation to LWSF programme

LWSF

- What are the key challenges/issues in the area you serve, in relation to smoking and reducing rates of smoking
  - What are the priority areas/groups (particular demographics / disadvantage / geography)
  - Barriers around these (access/costs)
  - What are current barriers around recruiting smokers (self referral/from smokers)
    - Current approaches used to recruit
- Can you briefly provide an explanation of how the current service (the hybrid LWSF programme) runs? – what is the current set up/offer?
  - Have there been any changes in recent years due to commissioning/budget cuts / priorities, etc.
- What is your understanding of why the hybrid programme was established / what was the rationale / background of why the hybrid programme was established?
  - Do you think the hybrid approach will help recruit more smokers?
- How effective do you think the hybrid programme is working in providing support for people trying to stop smoking?
  - Is it helping people engage
    - Have you seen different rates of engagement with the sessions?
  - It is helping people quit sooner and longer

- Is the current programme/approach working better for some groups? / is it accessing and engaging ‘target’ groups?
  - - For who
    - why/why not
    - are there key priority groups you work with? Is it working for them
  - What are the advantages and disadvantages of the different approaches (face-to-face, remote, blended - (for different populations)
    - Which groups benefit the most/least
    - Any groups not accessing/engaging
  - What choice/flexibility do patients get about what pathway/support they receive
    - Would providing more choice / more flexibility be a good/bad thing

- - Are you comfortable providing support remotely?
    - Is there training/technological needs associated with remote provision (+ supervision needs)
  - Are service users comfortable receiving support remotely?
    - From your experience, what are service users perspectives of the different pathways / what do they think about the hybrid model
- What are the **facilitators and barriers for you** delivering the programme?​
- What do you think are the **facilitators and barriers for people participating** in the programme?
  - - Accessibility
    - Convenience
    - Rapport /relationship building
    - Engagement/attendance
- What are the time/resources required (for the different pathways)
  - Is the programme an effective use of your/staff time/resources
    - more/less/same workload
    - less/more travel time (to offices – work/life balance)
    - Is more remote working a good thing (for you – for service users)
  - Do you think more people can be seen through remote/hybrid/blended provision

Reflections/future plans

- What could be improved in relation to the hybrid model?
- What have been some of the implementation/current issues of the programme?
- What would you like to see in relation to the LWSF programme going forward?
  - would you like this model to continue?
  - What approach/model would you prefer
  - What initial learning has been gained / and how has this been used

**Service user topic guide**

Introduction:

- Thank you for participating
- Introduction of self
- introduction of study
- Key points
- length of interview (30 minutes)
- interview as a discussion which will cover key topics
- no right or wrong answers - exploring perspectives
- participation is voluntary - rights around withdrawing participation
- confidentiality/anonymity
- how findings will be reported
- interview will be audio recorded
- Questions? Happy to proceed? Complete and sign consent form

Participant background

- How did you hear about the LWSF service / How did you come into contact with the LWSF service?
  - (self-referral - referred in by service)
- What were the main reasons why you decided to use the LWSF service

LWSF

- How was programme/support/treatment explained to you
  - What kind of treatment/support pathway did you receive
    - (face-to-face, remote, blended) / medication ?
  - Did you have any choice/flexibility in options of support
    - seen f-2-f, online/phone, mixture / Types of treatment/medication
    - What would you have preferred
- How helpful or unhelpful would you say the LWSF service was in helping you quit?
- What was your experience of the programme like (of receiving support via face-to-face, remote, blended)
  - Was it a useful way to get support
  - Is there anything that could have been done differently
  - Have you received smoking cessation support previously?
    - YES = Has the service been different than support you’ve had previously
    - NO = was it different to what you were expecting?
- What have been good/bad features of your support?
  - Accessibility / Convenience (time needed to attend / distance to travel)
  - Rapport building
  - Engagement/attendance (motivation to attend sessions)
    - Was it easy to access and take part in the support sessions
      - What made/could have made it easy/harder
    - Did it feel easy to get to and take park in sessions (time/travel/cost)
    - Did you attend all the sessions
    - How was it using the phone/video call / how was it attending face-to-face
      - Do you think this made a difference to how easy is was to interact with your worker/adviser (to develop a relationship)
      - Did the session feel private
      - Did you feel comfortable and able to communicate effectively
    - Did how you received support impact on your motivation/abilities to quit
      - Would support have been better if provided in a different way
- How effective do you think the hybrid programme is working in providing support for people trying to stop smoking
  - Are there any issues
    - how could these be overcome
- Overall, what are your thoughts about the programme
  - Are there any recommendations you would make for improving the service / What would you like to see changed
  - Would knowing more about the LWSF programme (e.g., what kinds of support you can receive (remote/medication) make you/other more likely to use the service?

Thank and finish

- anything else they would like to add

**Coding Framework**

**Revised coding framework**

| **Theme** | **Sub-theme one** | **Sub-theme two** |
| --- | --- | --- |
| Reach to priority groups | - Accessing needed groups/populations - Understanding of hybrid service purpose/process - Comparison to previous service delivery | - Service support offered/received - Knowledge of service process from service users |
| Perceived efficiency of the hybrid intervention | - Benefits/challenges of hybrid - Benefits/challenges of remote (phone/text/video) - Benefits/challenges of face-to-face | - Accessibility - Convenience/Comfort - Rapport /relationship building - Engagement/attendance - Privacy - CO testing |
| Accommodation of service users’ needs | - Effectiveness for different groups/populations (accessing and engaging) - Service users’ flexibility/choice around support |  |
| Implementation of the hybrid service | - Service time/resources requirements | - Service targets - Capacity and caseloads |
| Maintenance of the hybrid service | - Suggested improvements | - Service delivery - Service advertising |

**Initial coding framework**

| **Theme** | **Sub-theme one** | **Sub-theme two** |
| --- | --- | --- |
| Understanding of hybrid service purpose | - Reasons for hybrid development/initiation |  |
| Understanding of Hybrid service process | - Service support offered/received - Service users’ flexibility/choice around support - Knowledge of service process from service users - Comparison to previous service delivery |  |
| Perceived effectiveness of hybrid model | - Benefits/challenges of hybrid - Benefits/challenges of remote (phone/text/video) - Benefits/challenges of face-to-face | - Accessibility - Convenience/Comfort - Rapport /relationship building - Engagement/attendance |
|  | - Effectiveness for different groups/populations (accessing and engaging) |  |
| Service time/resources requirements |  |  |
| Suggested improvements | - Service delivery - Service advertising |  |

**Service user demographic information**

To protect participants confidentiality, we have not linked participant numbers to characteristics.

| **Gender** | **Age** | **Ethnicity** | **Provision** | **Other** |
| --- | --- | --- | --- | --- |
| Female | 25-34 | White British | Phone | Midwife referral |
| Male | 35-44 | White British | Phone | Self-referral |
| Female | 45-54 | White British | Phone | GP |
| Female | 25-34 | Mixed/Multiple ethnic group | Mixed/Hybrid | Midwife referral |
| Female | 35-44 | Asian/Asian British | Phone | GP |
| Female | 35-44 | White British | Face-to-face | Self-referral |
| Female | 55-64 | White British | Face-to-face | GP |
| Female | 35-44 | White British | Phone | Self-referral |
| Female | 55-64 | White British | Phone | GP |
| Female | 45-54 | Mixed/Multiple ethnic group | Phone | Self-referral |
| Male | 25-34 | White British | Phone | Self-referral |
| Female | 25-34 | White British | Phone | GP |
| Female | 35-44 | White British | Phone | Self-referral |
| Female | 35-44 | White British | Phone | GP |
| Female | 25-34 | White British | Phone | Midwives |
| Male | 55-64 | White British | Mixed/Hybrid | GP |
