# Supplementary material for: Reintroducing face-to-face support alongside remote support to form a hybrid stop smoking service in England: a formative mixed methods evaluation
Source: BMC Public Health. 2024 Mar 6;24:718. doi: 10.1186/s12889-024-18235-0 (PMC10916048; doi:10.1186/s12889-024-18235-0)
Supplement: Supplementary file 3 — Supplementary Material 3 [file 12889_2024_18235_MOESM3_ESM.docx]

**Supplementary material for “Reintroducing face-to-face support alongside remote support to form a hybrid stop smoking service in England: A formative mixed methods evaluation”**

Additional file 3

**Supplementary quantitative methods and results**

Summary of the data provided by the LWSF service around monitoring of the three support pathways

Working with our LWSF partners, descriptive statistics were produced on:

- The number and percentage of service users who chose each mode of contact, regardless of whether they subsequently set a quit date.
- The number and percentage of service users who set a quit date for each mode of contact.
- The number and percentage of service users who subsequently achieved a 4-week quit or were lost to follow-up.

Emerging data around variations in quit outcomes between the different pathways for different priority groups

To investigate service reach to priority groups deemed important for our LWSF service partners, the statistics on setting a quit date were subsequently disaggregated by for pregnant women, people with mental health conditions, and people with long-term physical health conditions.

In the data provided by the service for this evaluation (Table 2), there were similar 4-week quit outcomes for pregnant women (76.4%), people with mental health conditions (72.8%) and people with long-term physical health conditions (74.8%). There were some slight variations in quit outcomes between the different pathways for different priority groups (Table 2), e.g. a higher percentage of 4-week quits were achieved with face-to-face support (85.7%) compared to phone support (72.4%) for people with mental health conditions. However, the early stage of implementation of the hybrid service and the low sample sizes in some categories lead to high uncertainty in comparisons.

Cost calculation of return on investment for the face-to-face offering

We estimated that the additional cost of providing a face-to-face option in the period September 2022 to February 2023 was £692.49 per month. This cost was based primarily on the costs of hiring rooms for face-to-face clinics at 7 sites at a cost of £15 /room/day for an average of 4 days /month, giving an average cost of room hire of £60.18 /site/month (range £50 to £72), and a total cost of £421.29 across all sites. Added to the cost of room hire are the costs of SSA travel to the face-to-face venues at £203.40 /month, and the costs of parking at those venues at £67.80 /month. In the average month from September 2022 to February 2023, 149 people were referred to the LWSF service. Using the percentages from Table 1, the service could expect 3.97 of these people to achieve a 4-week quit using either the face-to-face or mixed pathways. Dividing the total cost by the total quits gives a cost per 4-week quit from the additional face-to-face option of £174.61. The figures presented in the main manuscript are rounded to the nearest whole number. Note that this does not include the costs of SSA time, CO monitoring, or other costs of running the service that are shared between remote and face-to-face provision.

* note that the numbers in the manuscript are rounded, the unrounded numbers are used here.

Reason for exclusion of CO monitoring in cost calculation:

In the original costing plan, we had suggested to cost the D-pieces and cardboard tubes, which are the consumable aspects of CO monitoring. However, on discussion with the service it was decided not to include these. This was due to the low rates of using the face-to-face option (it was not being used to capacity) and the consequently low rates of CO monitoring (current rate was about 5%), which means that the majority of the costs of providing a face-to-face service were accounted for by venue hire and staff travel expenses. It was however noted in discussions that if the use of the face-to-face option increased and this was done with CO monitoring (e.g. it could be that 90% of all quits need CO validation), then the additional costs of CO monitoring would increase and they should be taken into account. However, if this happens, then the costs of CO monitoring might apply to both the remote and face-to-face pathways (e.g. if someone who is remote has to come in for a face-to-face CO monitoring session). If this increase in CO monitoring happens, then the costs to the service of buying the CO monitoring consumables would need to be ascertained. However, given the relatively modest cost associated with CO monitoring, and the potential need for CO monitoring for both the remote and face-to-face options, then its inclusion in costs is unlikely to affect conclusions on the cost-effectiveness of offering face-to-face provision.
